# Supplementary material for: Development and implementation of a Digital Twin workshop for a smoke alarm production line
Source: PLoS One. 2026 Jan 29;21(1):e0341224. doi: 10.1371/journal.pone.0341224 (PMC12854460; doi:10.1371/journal.pone.0341224)
Supplement: Attachment — Submitted filename: Response to the Reviewers comments.docx [file pone.0341224.s002.docx]

**Dear Editor and Reviewers,**

We thank you very much for giving us another chance to revise and improve our manuscript. We try our best to revise the paper according to the reviewers’ suggestions, and for those comments we have different opinions, we have explained in detail. A list of changes and responses are listed below.

We hope the revised paper has addressed all the reviewers’ concerns and reached the requirements of the journal for publishing.

Thank you very much!

The authors

**Responses to Reviewers’ Comments**

**Reply to reviewer #1**

1. **Concern of the reviewer:**

**The literature review focuses on industrial applications of digital twins but does not mention case studies comparing related technologies in other low-automation industries (e.g., traditional manufacturing). It is recommended to include discussions of studies in similar contexts.**

**Our response:** We are grateful to the Reviewer for his elaborate work to find out our weakness.We have added the comparison between digital twin technology and traditional manufacturing .

**Revised text:**

At the same time, many scholars have made a comparative study of digital twin technology and traditional manufacturing technology. Semeraro, et al. [9] proposed a data-driven digital twin modeling method to improve the robustness of the model through the invariance mode, and compared with the flexibility and scalability of traditional rule-based modeling methods in complex production systems. Pronost, et al. [10] systematically reviewed the application of digital twins in the whole product life cycle (design, production, operation and maintenance), pointed out the limitations of traditional simulation technology in real-time and cross scale integration, and emphasized the advantages of digital twins in dynamic optimization. The case showed that the product development cycle was shortened by 28%. Mayer, et al. [11] analyzed the technical trend of digital twins in manufacturing, compared the limitations of traditional simulation tools in multi physical field coupling modeling, proposed the advantages of digital twins in interdisciplinary collaboration, and quantified its improvement in the modeling accuracy of complex systems by 42%. Fu Xiangfu, et al.[12] aimed at the limitations of traditional processing error research methods, proposed a digital twin driven multi-scale quality representation framework, and compared with the traditional off-line detection method, verified that its accuracy in real-time error compensation was improved by 51%. D'ambrogio, et al.[13] reviewed the application of AI driven digital twins in manufacturing, compared with the traditional threshold based maintenance strategy, and proposed the dynamic decision-making advantage of digital twins in predictive maintenance. The case showed that the downtime of equipment was reduced by 34%. Pathy, et al. [14] built a supply chain optimization model driven by digital twins, and compared with the traditional static planning method to verify its robustness under dynamic disturbance. The experiment showed that the logistics cost was reduced by 29%. Park, et al. [15] developed a digital twin driven optimization system for production line design. Compared with the limitations of traditional simulation software in layout design, the case shows that the reconstruction time of production line is reduced by 40%. Through specific cases and data comparison, the above papers have revealed the significant advantages of digital twin in design cycle, cost control, real-time optimization, and pointed out the shortcomings of traditional manufacturing technology in complex system management and dynamic response.

2. **Concern of the reviewer:**

**The description of digital twin model construction in Section 3 relies heavily on mathematical expressions for definitions. While these equations provide a theoretical framework, they are highly abstract, lacking derivations of variables and their applications, as well as specific details on how they are quantified or implemented in Siemens NX software. The authors should provide concrete examples and relevant descriptions.**

**Our response:** Thanks very much to the Reviewer for his meticulous work to find out our weakness , To solve the above problems, in order to make readers more clearly understand the production content of the smoke alarm base, we added the section on the physical system composition and process flow of the base production line, adjusted the original section 3 to section 4, and added the section 4.4 on the construction of NX twin model in section 4, which described in detail how to build the twin model in NX.

**Revised text:**

**3. Physical system composition and process flow of the base production line**

**3.1 Physical system composition**

The production line for smoke alarm bases comprises five core subsystems (as depicted in Figure 2). The equipment and functions of each subsystem are detailed below:

Injection Molding Subsystem: this includes one horizontal injection molding machine (with a clamping force of 500 KN), a raw material dryer, and a cooling water circulation system. It is responsible for melting, injecting, pressure-holding, and cooling the plastic raw materials to form the base.

Assembly Subsystem: comprising two six-axis robots (with a repeat positioning accuracy of ±0.02 mm), a conveyor belt (operating at a speed of 0.5 m/s), and tooling fixtures, this subsystem handles the assembly of the base and metal inserts.

Machine Vision Subsystem: this subsystem includes two industrial cameras (each with 5 million pixels), lenses (with a focal length of 16 mm), an annular LED light source, and an image processing unit. It is used for assembly positioning guidance and quality inspection.

Detection Subsystem: equipped with size detection sensors (with an accuracy of ±0.01 mm) and appearance defect detection devices, this subsystem conducts a comprehensive inspection of the assembled bases.

Packing and Sealing Subsystem: this subsystem includes an automatic packing machine, a tape sealing machine, and a labeling machine, which together facilitate the packaging and identification of qualified bases.

**Figure2 Physical drawing of workshop**

**3.2 Process flow analysis**

The entire production process of the base can be categorized into four crucial stages, with the following process parameters and logical relationships for each:

Injection Molding Stage: raw materials undergo drying (at a temperature of 80°C for 4 hours) before being fed into the injection molding machine's barrel. Upon heating (with a nozzle temperature of 220°C), they melt and are injected into the mold cavity. The pressure is maintained at 80 MPa for 3 seconds, followed by a cooling period of 15 seconds. Once the mold is opened, the base blank is ejected.

Manipulator Assembly Stage: the conveyor belt transports the base blank to the assembly station, where the machine vision system captures an image of the base's positioning holes. After calculating any deviations, the manipulator is guided to grasp the metal insert (with a diameter of 8 mm) and press it into the preset position on the base (with a pressing force of 500 N).

Visual Inspection Stage: an industrial camera at the inspection station captures 360° images of the assembled base. Through image algorithms (combining template matching and edge detection), it assesses the perpendicularity of the insert assembly (with a tolerance of ±0.1 mm) and detects any appearance defects, such as cracks or material shortages.

Packing and Sealing Stage: qualified bases are conveyed to the packing machine via a conveyor belt and stacked in boxes of 20 each. After the sealing machine applies tape to the top and bottom of the box, a labeling machine prints and affixes a QR code label containing the production date and batch information. Each stage is logically controlled by a PLC (Siemens S7-1200), with state feedback provided by sensors (proximity switches and pressure sensors), thereby forming a continuous production process.

**4.4 Construction based on NX twin model**

The construction of the twin model for the smoke alarm base production line adheres to a hierarchical approach of "equipment level → unit level → production line level," with the specific framework outlined below:

Equipment Level Model: establish geometric and behavioral models for individual equipment such as injection molding machines, manipulators, and cameras.

Unit Level Model: integrate equipment-level models into subsystems (e.g., injection molding, assembly) to facilitate collaborative simulation among equipment within these subsystems.

Production Line Level Model: combine unit-level models to create a virtual representation of the entire production line, enabling comprehensive process simulation and optimization.

he modeling process must adhere to the three principles of "geometric consistency, behavioral equivalence, and parameter correlation" to guarantee that the virtual model accurately mirrors the shape, motion, and performance of the physical entity.

**4.4.1 Equipment level model construction**

(1) Modeling of injection molding machine

Geometric modeling:

Using NX's "synchronous modeling" technology, the 3D skeleton model is generated by importing the 2D drawing (CAD format) of the injection molding machine.

Decompose the injection molding machine into sub components such as clamping mechanism, injection mechanism and ejection mechanism, and use "Boolean operation" and "feature modeling" (such as stretching and rotation) to build the detail structure.

The accuracy of key components (such as screw and mold cavity) is controlled at 0.005mm, and non key components (such as shell) can be simplified (removing chamfer, small hole and other features) to reduce the complexity of the model.

Behavior modeling:

Define the motion pair in NX MCD: add "rotation pair" (opening and closing mold rotation axis) and "translation pair" (template movement) to the clamping mechanism, and add "screw pair" (screw rotation and axial movement) to the injection mechanism.

Associated driving parameters: drive the motion pair through the "servo motor", set the mold opening and closing speed (0.5m/s), injection pressure (0-100Mpa) and other parameters, corresponding to the PLC control parameters of the physical equipment.

Add sensor model: add "position sensor" (to detect the opening and closing state of the mold) and "pressure sensor" (to simulate the cavity pressure) at the mold cavity position, and its signal output is bound to the state variable of the virtual model.

(2) Manipulator and machine vision model construction

Geometric modeling of manipulator:

The "top-down" design method is adopted. Firstly, the base and joint skeleton of the manipulator are constructed, and then the arm, wrist and end effector are added through "assembly constraints" (such as coincidence and parallel).

The end effector (gripper) adopts parametric design, and the opening and closing size of the gripper is associated through "expression" (to adapt to inserts with a diameter of 5-10mm).

Robot behavior modeling:

Add a "rotation pair" at the joint to set the rotation range (-180 °~180 °) and angular velocity (50 °/s).

Through the "path planning" function of NX, predefine the motion tracks of grabbing, moving and placing (adopt arc transition to avoid emergency stop).

Associate machine vision signals: install a virtual camera model at the wrist, set the field of view angle (60 °) and resolution (5million pixels), and the output "positioning deviation value" is used as the compensation parameter of the manipulator movement.

Machine vision system modeling:

Build the geometric model of camera, lens and light source in NX, and define the installation position through the "coordinate system" (consistent with the installation coordinates of the physical camera).

Develop virtual image processing module: call opencv algorithm library through NX open API, realize "template matching" (locating the hole position of the base) and "defect detection" (simulating gray value analysis) in the virtual environment, and output the detection results (ok/ng signal).

(3) Construction of packing and sealing equipment model

Geometric modeling:

The sealing machine is decomposed into conveying roller table, pressing roller and belt cutting mechanism, and the "sheet metal design" module is used to build a thin shell structure (1mm thick).

The carton model adopts "folding design" to simulate the deformation characteristics of the carton through the "flexible body" attribute.

Behavior modeling:

Add "rotating pair" to the conveying roller table and set the speed (100r/min).

The "translation pair" is added to the pressing roller mechanism, and the pressing stroke (0-50mm) is set, and the pressure parameter (100N) is associated with the virtual pressure sensor.

The belt cutting mechanism simulates the cutting action through "Boolean operation" and sets the cutting delay time (consistent with the action time of the solenoid valve of the physical equipment).

**4.4.2 production line level model integration**

Layout integration:

In the "assembly" environment of NX, each equipment model is placed according to the actual layout of the physical workshop (the coordinate origin is aligned with the workshop ground reference point).

Ensure the relative position accuracy (± 5mm) between the equipment through "distance constraint" and "angle constraint", for example, the docking gap between the injection molding machine and the conveyor belt is set to 10mm.

Logical Integration:

The "signal flow" module of NX MCD is used to define the control signal between the equipment: the signal from the top of the injection molding machine triggers the start of the conveyor belt, and the camera at the signal trigger detection station is photographed after the assembly is completed.

Build virtual PLC logic: write control program in NX through "ladder diagram" to simulate the i/o point of physical PLC (for example, i0.0 is the ready signal of injection molding machine and q0.1 is the start signal of manipulator).

Performance Optimization:

Carry out "lightweight processing" on the model: delete invisible parts (such as internal cables of equipment), simplify surfaces (replace non critical surfaces with planes), and control the total number of triangular patches of the model within 5million;

Adopt "hierarchical display" technology: automatically load/unload model details according to the simulation perspective to improve the real-time rendering efficiency (the frame rate is kept above 30fps).

The final result is shown in Figure 5.

**Figure 5 Workshop digital twin model**

3. **Concern of the reviewer:**

**In Section 4, the case study validation mentions cost savings (2.1 million yuan/year) and a 2% output increase but lacks control group data (e.g., comparisons with traditional production lines) and corresponding details. It is suggested to include details of the experimental design and statistical analysis methods.**

**Our response:** We are grateful to the Reviewer for his elaborate work to find out our weakness .We added chapter 7.2 operation effect to introduce the verification and comparison between digital twin model and traditional production in detail and adjusted the data.

**Revised text:**

**7.2 Operation effect**

(1) Twin model accuracy verification

Geometric mapping accuracy: randomly select 10 base parts and measure the key dimensions (such as diameter and height) of the physical parts and the virtual model, with an average error of 0.08mm, meeting the design requirements (≤ 0.1mm).

Behavior synchronization accuracy: record the physical execution time and virtual simulation time of the manipulator grasp action, and the synchronization error is 35ms, which is lower than the threshold (50ms).

(2) Productivity improvement

Virtual debugging cycle: traditional physical debugging takes 72 hours, while virtual debugging based on digital twin takes only 24 hours, with an efficiency increase of 66.7%;

Equipment utilization rate: through real-time monitoring and scheduling optimization of virtual and real mapping, the equipment utilization rate increased from 85% to 92%, and the daily output increased to 5300 pieces.

(3) Quality and cost optimization

Quality inspection: the machine vision system improved the accuracy of defect recognition from 96% to 99.2% by optimizing the parameters of the virtual model;

Energy consumption reduction: optimization of injection molding process parameters based on virtual model (e.g. pressure holding time is shortened from 3 seconds to 2.5 seconds), and energy consumption per unit product is reduced by 8%.

4. **Concern of the reviewer:**

**Figures 7 and 8 are critical for demonstrating the system's actual implementation, but the text lacks descriptions of specific metrics in the interface (e.g., production progress, equipment status). For example, providing sample outputs or screenshots to illustrate the interface's functionality would be helpful. The authors should provide detailed information explaining the key elements depicted in each figure.**

**Our response:** We are grateful to the Reviewer for his elaborate work to find out our weakness.

We adjusted Figure 7 and figure 8 to figure 8 and Figure 9, and added a detailed description of figure 8 and Figure 9.

. **Revised text:**

By utilizing the twin model modeling approach, data acquisition and transmission solutions outlined in this paper, along with the process flow of the actual production line,we have developed a digital twin model for the smoke alarm base production line and established a virtual workshop. Powered by real-time data, the virtual workshop precisely mirrors the production and processing activities in the physical workshop.Building on this foundation,we have deployed and implemented a three-dimensional, visually enhanced real-time monitoring system in the workshop, leveraging digital twin technology, to enable real-time tracking of production status and assist staff in comprehensively understanding the workshop's production status and operational information. Figure8 displays the main interface of the twin system.

There are four main buttons at the top of the main interface, i.e. start, reset, pause and stop, which can manually control the start, pause, stop, reset and other functions of the production line. There is a current alarm display column under the four buttons. If the production line fails, it will scroll through the interface. The real-time output is displayed on the left side of the middle position of the main interface in real time. The simulation operation module can simulate key unit operations such as injection molding, assembly, packaging, and reclaiming before actual production. The right reset initialization status module manually initializes the main nodes of the production line process. At the bottom of the main interface, there are five button modules: manual page, alarm page, parameter page, debugging page and main page. The manual page can realize the function of manually operating the production line, the parameter page can display the production parameters of each equipment of the production line in real time, and the debugging page can debug each unit of the production line.

**Figure8 Twin system main interface**

Figure 9 shows the workshop monitoring interface. The middle position of the interface displays the working data of the production line in real time, and the corresponding equipment position displays the working status of the equipment in real time. Green indicates normal operation, yellow indicates standby, and red indicates fault alarm. The upper left corner of the interface displays the humidity, temperature, startup rate, gas supply pressure, utilization rate and other data of the workshop in real time. The upper right corner can display the alarm information of the production line in real time. An emergency stop button is set at the lower part of the interface, which can be manually stopped in case of emergency. The machine vision detection information that cannot be seen in the model is displayed at the bottom of the interface in real time. At the bottom right of the interface is the workshop data summary chart, which can display the workshop running time, product qualification rate summary, workshop output and other data in real time.

**Figure 9 System monitoring interface**

5. **Concern of the reviewer:**

**The figures provided in the article are mostly conceptual, focusing on architecture and models, with a lack of actual data and real-world scenarios.**

**Our response:** We are grateful to the Reviewer for his elaborate work to find out our weakness.

We added Chapter 3 to describe the real scene, added chapter 4.4 to describe how to carry out workshop modeling in NX, added Chapter 5 to describe how to carry out data collection and data transmission between the model and physical entities, and added Chapter 7 to describe the application scenario in detail, and compared the digital twin technology with traditional production.

**Revised text:**

**3. Physical system composition and process flow of the base production line**

**3.1 Physical system composition**

The production line for smoke alarm bases comprises five core subsystems (as depicted in Figure 2). The equipment and functions of each subsystem are detailed below:

Injection Molding Subsystem: this includes one horizontal injection molding machine (with a clamping force of 500 KN), a raw material dryer, and a cooling water circulation system. It is responsible for melting, injecting, pressure-holding, and cooling the plastic raw materials to form the base.

Assembly Subsystem: comprising two six-axis robots (with a repeat positioning accuracy of ±0.02 mm), a conveyor belt (operating at a speed of 0.5 m/s), and tooling fixtures, this subsystem handles the assembly of the base and metal inserts.

Machine Vision Subsystem: this subsystem includes two industrial cameras (each with 5 million pixels), lenses (with a focal length of 16 mm), an annular LED light source, and an image processing unit. It is used for assembly positioning guidance and quality inspection.

Detection Subsystem: equipped with size detection sensors (with an accuracy of ±0.01 mm) and appearance defect detection devices, this subsystem conducts a comprehensive inspection of the assembled bases.

Packing and Sealing Subsystem: this subsystem includes an automatic packing machine, a tape sealing machine, and a labeling machine, which together facilitate the packaging and identification of qualified bases.

**Figure2 Physical drawing of workshop**

**3.2 Process flow analysis**

The entire production process of the base can be categorized into four crucial stages, with the following process parameters and logical relationships for each:

Injection Molding Stage: raw materials undergo drying (at a temperature of 80°C for 4 hours) before being fed into the injection molding machine's barrel. Upon heating (with a nozzle temperature of 220°C), they melt and are injected into the mold cavity. The pressure is maintained at 80 MPa for 3 seconds, followed by a cooling period of 15 seconds. Once the mold is opened, the base blank is ejected.

Manipulator Assembly Stage: the conveyor belt transports the base blank to the assembly station, where the machine vision system captures an image of the base's positioning holes. After calculating any deviations, the manipulator is guided to grasp the metal insert (with a diameter of 8 mm) and press it into the preset position on the base (with a pressing force of 500 N).

Visual Inspection Stage: an industrial camera at the inspection station captures 360° images of the assembled base. Through image algorithms (combining template matching and edge detection), it assesses the perpendicularity of the insert assembly (with a tolerance of ±0.1 mm) and detects any appearance defects, such as cracks or material shortages.

Packing and Sealing Stage: qualified bases are conveyed to the packing machine via a conveyor belt and stacked in boxes of 20 each. After the sealing machine applies tape to the top and bottom of the box, a labeling machine prints and affixes a QR code label containing the production date and batch information. Each stage is logically controlled by a PLC (Siemens S7-1200), with state feedback provided by sensors (proximity switches and pressure sensors), thereby forming a continuous production process.

**4 Construction of workshop digital twin model**

The twin model of the workshop production process is the foundation for building a digital twin workshop, and it is also a true mapping of the physical workshop.The key elements of the workshop production process are people,machines,objects,environment,etc.According to the composition and production process of the smoke alarm production line workshop, the twin model of the workshop is divided into unit level, production line level, and workshop level.Unit level includes equipment twin models, personnel models, environmental models, and product/material models.At the production line level,fusion and assembly are achieved by adding spatial relationships, constraint relationships, and other related relationships to the unit level.Workshop level refers to the interconnection, interoperability, and collaborative optimization across systems through information flow, energy flow, and other means, based on the production line level.

To construct a digital twin model of the workshop as shown in Figure 3, the first step is to map the physical entity's dimensions, shape, assembly relationships, and other attributes and rules, so that the model can truly map the geometric and physical attributes of the smoke alarm production workshop based on known rules and attributes.Secondly, the behavior of physical entities is expressed in the model, and multidisciplinary, multi physical quantity, and multi-scale data related to production behavior such as mechanical data, electrical data, process control data, and functional description data are obtained through communication data interfaces.Finally, an information model is constructed to obtain massive data from the physical and model layers through data collection and virtual simulation. By comparing and analyzing the data between the physical and virtual workshops, decisions are made based on the relationships and rules between various parameters, and feedback and guidance are provided to the physical workshop to achieve optimized control of the entire production activity.Therefore, each model is a construction of fundamental models in multiple fields such as mechanics, electronics, and hydraulics, as well as multidimensional models of geometry, physics, behavior, and rules. The mathematical expression for constructing the twin model of the smoke alarm workshop is equation (1):

$WTM_{sa}=\left\{G_{sa},P_{sa},B_{sa},DI\right\}$ （1）

In the formula, *WTM_sa_* is the twin model of the smoke alarm base production workshop, *G_sa_* is the geometric model, *P_sa_* is the physical model, *Bsa* is the behavioral model, and *DI* is the data information model.

**Figure3 Modeling process of twin workshop for smoke alarm base production line**

**4.1 Geometric model**

The geometric model serves as a three-dimensional representation that delineates the shape, size, position, and assembly relationship of a physical entity, maintaining excellent temporal and spatial coherence with its real-world counterpart. A tree-structured chain is established based on the structural relationships within the smoke alarm base production workshop, as illustrated in Figure 4. With the smoke alarm base production workshop serving as the root node, it is further divided into four modules according to the workshop's model relationships: the equipment model, personnel model, material model, and environment model. Each node is then individually assigned a local coordinate system to store the positional coordinates relative to its parent node. In Siemens NX software, the components of each submodule are meticulously drafted at a 1:1 scale, assembled, and integrated based on assembly and parent-child relationships to form individual submodule models. These submodule models are then arranged according to the actual workshop layout to create a preliminary workshop model. To enhance the efficiency of computer graphics processing and simulation, the three-dimensional models undergo lightweight processing during modeling, minimizing unnecessary lines and surfaces. For components that do not significantly contribute to the simulation, it suffices to depict their overall dimensions and accurate motion patterns, without the need to represent other ancillary structures. The mathematical formulation for constructing the geometric model is provided as (2):

$G_{\text{sa}}=\{S_{\text{g}},D_{g},P_{g},AR_{g},PR_{g}\}$ （2）

In the formula: *S*_g_ is the geometric shape, *D*_g_ is the geometric dimension, *P*_g_ is the geometric position, *AR*_g_ is the assembly relationship, and *PR*_g_ is the positional relationship [11].

**Figure4 Tree diagram of smoke alarm base production workshop model**

**4.2 Physical model**

The primary function of a physical model is to delineate the physical attributes of equipment, materials, and personnel, encompassing mechanical, thermal, and optical properties of devices like injection molding machines, machine vision systems, robotic arms, and more. By incorporating physical attributes such as materials, mass, friction, and collision bodies into the geometric model, the physical characteristics of the equipment are realized. Furthermore, the physical model is constructed by integrating various sensors that capture real-time data on material movement and environmental parameters. The mathematical representation of this physical model is denoted as (3):

$P_{sa}=\{E_{p},M_{p},P_{p}PC,EP,\text{Info\}}$ （3）

In the formula, *E_p_* is the physical attribute of the equipment, *M_p_* is the physical attribute of the material, *P_p_* is the physical attribute of the personnel, *PC* is the physical characteristic of the equipment, *EP* is the environmental parameter, and Info is other information.

The physical attributes of equipment mainly include type number, element name, physical parameters, and production and processing status.Among them, the type number is the identification of all devices; Element names are used to distinguish between different digital twin models of the same manufacturing equipment abstract model;The physical parameters mainly include transmission speed, cutting force, maximum capacity and other data, and the physical parameters of different devices are different;The production and processing status refers to the state at different times during the operation of the equipment. The mathematical expression is (4):

$E_{p}=\left\{TM,Name,PP,MSS\right\}$ （4）

In the formula, *TM* is the device type number, *Name* is the feature name, *PP* is the physical parameter of the device, and *MSS* is the model state set.

The physical properties of materials mainly include material name, material variety, material model, material status, etc. The mathematical expression is (5):

$M_{p}=\left\{M_{name},M_{v},M{}_{t},M_{s}...\right\}$ （5）

In the formula, *M_name_* is the material name, *M_v_* is the material variety, *M_t_* is the material model, and *M_s_* is the material status.

The physical attributes of personnel mainly include personnel number, personnel name, personnel occupation, personnel shift, etc. The mathematical expression is (6):

$P_{p}=\left\{P_{i},P_{n},P_{w},P_{c}...\right\}$ （6）

In the formula, *Pi* personnel number, *_Pn_* personnel name, *Pw* personnel job type, *_Pc_* personnel shift.

The physical properties of the environment include temperature, humidity, illumination, air pressure, etc. The mathematical expression is (7):

$EP=\left\{EP_{tem},EP_{hum},EP_{I},EP_{p}...\right\}$ （7）

In the formula, *EP_tem_* is the ambient temperature, *EP_hum_* is the ambient humidity, *EP_I_* is the illumination, *EP_P_* is the air pressure.

**4.3 Behavioral model**

The behavior model primarily serves to delineate the production processes of the smoke alarm base, encompassing the injection molding of the shell from raw materials within a temporal framework and internal operational mechanisms, followed by the assembly of components, quality inspection, and packaging. The behavior models are categorized into sensor modeling and actuator modeling [12]. The operational parameters of the equipment are configured based on specified requirements, with the corresponding sensors on the production line (including temperature and humidity sensors, pressure sensors, displacement sensors, flow sensors, speed sensors, position sensors, vision sensors, etc.) being defined according to their operational principles. Through sensor behavior modeling, signals emanating from the digital twin model of the production line, based on operational conditions, are relayed back to the control system. To enhance the precision of triggering the transition from one process stage to the next and mitigate errors arising solely from computational triggers meeting criteria, position sensors are installed at the entry and exit points of each process stage. The operational timing of the equipment is determined by a combination of factors: the calculation of operational conditions, the triggering of outlet sensors by the preceding batch of products, and the triggering of inlet position sensors by the subsequent batch of products. This approach enhances the accuracy and success rate of production.

Based on the working principle of driving the corresponding equipment, define the motion control parameters of the corresponding executing elements, and associate them with the interaction signals of the control system and the motion attributes of the digital model. With the help of actuator behavior modeling, the digital model can realize the motion of the executing mechanism driven by the output signal of the control system [13].

The data during the production process of smoke alarm base includes process parameter set *C*_parm_, process sequence set *P*_ss_, equipment operation dataset *D*_data_, part operation dataset *A*_data_, behavior coupling relationship set *BCR*, basic event set *BE*, production line environment parameter set *EP*, and other information *Info*, which are obtained and rendered onto the model through the data interface set. Therefore, the behavior model of the smoke alarm base production line can be expressed in mathematical language as equation (8):

$B_{\text{sa}}=\text{（}C_{\text{parm}},P_{ss},D_{data},A_{data},BCR,BE,EP,Info...)$ （8）

**4.4 Construction based on NX twin model**

The construction of the twin model for the smoke alarm base production line adheres to a hierarchical approach of "equipment level → unit level → production line level," with the specific framework outlined below:

Equipment Level Model: establish geometric and behavioral models for individual equipment such as injection molding machines, manipulators, and cameras.

Unit Level Model: integrate equipment-level models into subsystems (e.g., injection molding, assembly) to facilitate collaborative simulation among equipment within these subsystems.

Production Line Level Model: combine unit-level models to create a virtual representation of the entire production line, enabling comprehensive process simulation and optimization.

he modeling process must adhere to the three principles of "geometric consistency, behavioral equivalence, and parameter correlation" to guarantee that the virtual model accurately mirrors the shape, motion, and performance of the physical entity.

**4.4.1 Equipment level model construction**

(1) Modeling of injection molding machine

Geometric modeling:

Using NX's "synchronous modeling" technology, the 3D skeleton model is generated by importing the 2D drawing (CAD format) of the injection molding machine.

Decompose the injection molding machine into sub components such as clamping mechanism, injection mechanism and ejection mechanism, and use "Boolean operation" and "feature modeling" (such as stretching and rotation) to build the detail structure.

The accuracy of key components (such as screw and mold cavity) is controlled at 0.005mm, and non key components (such as shell) can be simplified (removing chamfer, small hole and other features) to reduce the complexity of the model.

Behavior modeling:

Define the motion pair in NX MCD: add "rotation pair" (opening and closing mold rotation axis) and "translation pair" (template movement) to the clamping mechanism, and add "screw pair" (screw rotation and axial movement) to the injection mechanism.

Associated driving parameters: drive the motion pair through the "servo motor", set the mold opening and closing speed (0.5m/s), injection pressure (0-100Mpa) and other parameters, corresponding to the PLC control parameters of the physical equipment.

Add sensor model: add "position sensor" (to detect the opening and closing state of the mold) and "pressure sensor" (to simulate the cavity pressure) at the mold cavity position, and its signal output is bound to the state variable of the virtual model.

(2) Manipulator and machine vision model construction

Geometric modeling of manipulator:

The "top-down" design method is adopted. Firstly, the base and joint skeleton of the manipulator are constructed, and then the arm, wrist and end effector are added through "assembly constraints" (such as coincidence and parallel).

The end effector (gripper) adopts parametric design, and the opening and closing size of the gripper is associated through "expression" (to adapt to inserts with a diameter of 5-10mm).

Robot behavior modeling:

Add a "rotation pair" at the joint to set the rotation range (-180 °~180 °) and angular velocity (50 °/s).

Through the "path planning" function of NX, predefine the motion tracks of grabbing, moving and placing (adopt arc transition to avoid emergency stop).

Associate machine vision signals: install a virtual camera model at the wrist, set the field of view angle (60 °) and resolution (5million pixels), and the output "positioning deviation value" is used as the compensation parameter of the manipulator movement.

Machine vision system modeling:

Build the geometric model of camera, lens and light source in NX, and define the installation position through the "coordinate system" (consistent with the installation coordinates of the physical camera).

Develop virtual image processing module: call opencv algorithm library through NX open API, realize "template matching" (locating the hole position of the base) and "defect detection" (simulating gray value analysis) in the virtual environment, and output the detection results (ok/ng signal).

(3) Construction of packing and sealing equipment model

Geometric modeling:

The sealing machine is decomposed into conveying roller table, pressing roller and belt cutting mechanism, and the "sheet metal design" module is used to build a thin shell structure (1mm thick).

The carton model adopts "folding design" to simulate the deformation characteristics of the carton through the "flexible body" attribute.

Behavior modeling:

Add "rotating pair" to the conveying roller table and set the speed (100r/min).

The "translation pair" is added to the pressing roller mechanism, and the pressing stroke (0-50mm) is set, and the pressure parameter (100N) is associated with the virtual pressure sensor.

The belt cutting mechanism simulates the cutting action through "Boolean operation" and sets the cutting delay time (consistent with the action time of the solenoid valve of the physical equipment).

**4.4.2 production line level model integration**

Layout integration:

In the "assembly" environment of NX, each equipment model is placed according to the actual layout of the physical workshop (the coordinate origin is aligned with the workshop ground reference point).

Ensure the relative position accuracy (± 5mm) between the equipment through "distance constraint" and "angle constraint", for example, the docking gap between the injection molding machine and the conveyor belt is set to 10mm.

Logical Integration:

The "signal flow" module of NX MCD is used to define the control signal between the equipment: the signal from the top of the injection molding machine triggers the start of the conveyor belt, and the camera at the signal trigger detection station is photographed after the assembly is completed.

Build virtual PLC logic: write control program in NX through "ladder diagram" to simulate the i/o point of physical PLC (for example, i0.0 is the ready signal of injection molding machine and q0.1 is the start signal of manipulator).

Performance Optimization:

Carry out "lightweight processing" on the model: delete invisible parts (such as internal cables of equipment), simplify surfaces (replace non critical surfaces with planes), and control the total number of triangular patches of the model within 5million;

Adopt "hierarchical display" technology: automatically load/unload model details according to the simulation perspective to improve the real-time rendering efficiency (the frame rate is kept above 30fps).

The final result is shown in Figure 5.

**Figure 5 Workshop digital twin model**

**5 Data acquisition and transmission**

**5.1 Workshop information system architecture**

The core of digital twin is modeling and data interaction. The design of a reasonable digital twin workshop information physical fusion system is the basis for the construction of virtual real interaction in digital workshop, and also the key to solve the problem of information island in digital workshop.In the production workshop for smoke alarm bases, there are numerous field devices, each with its unique protocol.To address this, a unified data model is necessary to facilitate data transmission and retrieval.The workshop has integrated OPC UA servers into each intelligent device on the production line. By utilizing information modules to analyze data collected from the underlying devices, these devices are connected to nodes within the OPC UA server's address space. Interaction for big data analysis is achieved through the connection between OPC UA server endpoints.The OPC UA information network structure comprises three layers: the equipment layer, the communication layer, and the information layer. The underlying equipment primarily includes production equipment, auxiliary devices, and sensors. At the equipment layer, operational and status data generated by the normal operation of the equipment are first encapsulated in the OPC UA server. Subsequently, this data is transmitted to higher-level applications using OPC UA's subscription/publish or query modes. Devices can directly communicate with the OPC UA server through OPC UA clients. The communication layer serves as the bridge between underlying facilities and information systems, as well as external networks and cloud applications. At the information layer, the OPC UA client is used to obtain various data from the equipment layer and store it in a local database for use by resource planning (EPR) and manufacturing execution systems (MES). Figure6 illustrates the workshop's information system architecture.

**Figure6 The workshop's information system architecture**

**5.2 Data acquisition system design**

**5.2.1 Types and parameters of collected data**

The data to be collected by the substructure production line is divided into four categories, and the specific parameters are as follows（shown in Table 1）:

**Table 1. Data acquisition parameters**

| **Data type** | **Collection object** | **Key parameter** | **Acquisition frequency** | **Accuracy requirements** |
| --- | --- | --- | --- | --- |
| Equipment status data | Injection molding machine, manipulator, etc | Operation status (operation/shutdown/fault), motor speed, temperature | 100ms | State recognition accuracy 100% |
| Process parameter data | Injection molding machine | Injection pressure, holding time, mold temperature | 50ms | ±0.1MPa（pressure） |
| Quality inspection data | Machine vision system | Positioning deviation, defect type, dimension measurement value | 1s/time (per product) | ±0.01mm（size） |
| Logistics information data | Conveyor belt , sealing machine | Base position, conveying speed, packing quantity | 200ms | position error ±5mm |

**5.2.2 Hardware acquisition architecture**

Sensor layer:

Various sensors are installed on the physical equipment: pressure sensor (range 0-200mpa) and temperature sensor (PT100, -50~200 ℃) are installed on the injection molding machine.

The encoder (resolution 1024 lines) installed on the conveyor belt is used for speed detection, and the laser displacement sensor (accuracy ± 0.001mm) is installed at the assembly station to detect the height of the insert.

Edge layer:

The industrial gateway (such as Siemens IOT 2050) is used as the data acquisition node, and the PLC is connected through PROFINET bus to obtain the equipment control signal.

OPC UA server (such as kepserverex) is built in the gateway to convert sensor and PLC data into OPC UA protocol format.

Transport layer:

Industrial Ethernet (Gigabit bandwidth) is used to build the communication network. The equipment and gateway are connected by wire (tcp/ip protocol), and wireless backup (5g) is supported between the gateway and the server.

The network delay is controlled within 50ms, and the "priority queue" ensures the priority transmission of key data (such as fault signals).

**5.3 OPC UA information model construction**

**5.3.1 Information model construction process**

The OPC UA based information model needs to map the physical entity and data relationship of the base production line. The specific construction steps are as follows:

(1) Namespace definition

Define custom namespace (URI: http://base-production-line.com/opcua ）, distinguished from OPC UA standard namespace, Namespace contains object nodes such as "production line", "production unit" and "assembly unit".

(2) Object level design

Root node: baseproductionline.

First level child nodes: injectionunit (production unit), assemblyunit (assembly unit), inspectionunit (inspection unit), packingunit (packaging unit).

Secondary child nodes: each unit contains specific equipment objects (such as injectionmoldingmachine, robot, camera, etc.).

Three level child nodes: the device object contains variables and method nodes such as status, parameters, and operation (such as the injectionpressure variable and the start method).

(3) Variable node attribute definition

Take the "injection pressure" variable of the injection molding machine as an example, define the attributes（shown in Table 2）:

**Table 2. Variable node attribute definition**

| **Data type** | Float |
| --- | --- |
| **Engineering unit** | MPa |
| **Minimum value** | 0 |
| **Maximum value** | 100 |
| **Access rights** | Readable and writable (support the virtual model to adjust the parameters of physical devices |
| **Sampling interval** | 50ms |

**5.3.2 Information Model for the Base Production Line**

On the organizational structure, functional modules, and data flow requirements among these modules in the smoke alarm base production workshop, the physical equipment and production process data within the workshop are digitally described and modeled, organized according to information modeling principles to form the digital workshop information model for smoke alarm base production. This model encompasses a static attribute set, a process attribute set, and a functional component set. The workshop information model aligns with the entire workshop, the functional component set corresponds to each functional module within the workshop, and the resource component set represents the various resources involved in each module. The attribute set signifies a collection of attributes, which serve as the fundamental unit of description in the information model. With a fixed structure, attributes are used to delineate the associations among devices, components, attribute sets, and attributes themselves.

The production line for smoke alarm bases boasts a variety of equipment types, posing significant integration challenges. The integration of this equipment and the acquisition of data are crucial to the performance of digital workshops.The OPC Unified Architecture(OPC UA) offers a standardized communication mechanism that can be synchronous, asynchronous, or distributed. This mechanism allows for the access of different types of data both horizontally and vertically. Based on OPC UA, this paper constructs an information model for the digital twin workshop of the smoke alarm base production line, as illustrated in Figure 7. The model comprises a static attribute set, a process attribute set, and a functional component set. The static attribute set corresponds to the fundamental static information of workshop operations, encompassing basic workshop information (such as the workshop's name, location, area, and person in charge), production organization (such as the departments and workgroups within the workshop), the work calendar (such as working days and rest days), and production work orders (order information obtained by the MES system from the ERP system) [14]. The process attribute set corresponds to the dynamic data of workshop processes, typically comprising aggregated statistical data from various aspects of the workshop and order tracking information, which is used to provide feedback to the ERP system. This includes order tracking, production statistics, quality statistics, inventory statistics, and maintenance statistics[15].The functional component set aligns with various production type modules within the production workshop, encompassing units for production, assembly, quality inspection, packaging, among others. Each production type module comprises a static attribute set, a procedural attribute set, and a resource component set. The static attribute set denotes the fundamental information pertinent to each module, including work plans, production techniques, and shift outputs. The procedural attribute set represents the production process information for each resource module, encompassing production plan tracking, operational data, and production statistics. The resource component set signifies the diverse resources associated with each module, such as injection molding machines, industrial robots, and quality inspection equipment.

**Figure7 Information model of digital production workshop for smoke alarm base production line**

**5.4 Data transmission realization**

The integration of NX and OPC UA serves as the pivotal aspect for enabling digital twin virtual-to-real data interaction within the base production line. By configuring an OPC UA client within the NX environment, establishing mechanisms for data subscription and publication, and devising strategies for handling exceptions, a real-time data pathway between virtual models and physical entities is established. This integration solution must fulfill the criteria of industrial-grade real-time performance (with a data update delay of ≤100ms), security (preventing data leakage and tampering), and reliability (ensuring no single point of failure). The detailed implementation process is outlined as follows:

(1) OPC UA client configuration in NX

First, enable the "OPC UA client" module in the NX MCD, connect the OPC UA server of the edge gateway through the "server address", then configure the security policy, use the "basic256sha256" encryption algorithm, and import the server certificate to complete the identity authentication.

(2) Data subscription and publishing mechanism

First, the virtual model subscribes to the physical device data.Associates variables (such as the "injection pressure" of the virtual injection molding machine) in NX with the corresponding node of OPC UA server (ns=1; s=injectionunit.injectionmoldingmachine.injectionpressure), and sets the subscription cycle of 50ms. Secondly, the virtual model issues control instructions. When adjusting parameters (such as manipulator speed) through virtual debugging, the NX client publishes the new parameter values to the "robot.speed" node of the OPC UA server, and the physical PLC acquires and executes the instructions by subscribing to this node.

(3) Data Exception Handling

Firstly, we adopt the "heartbeat detection" mechanism. If no data is received from the physical device within 100 milliseconds, NX automatically labels the data as "invalid" and triggers an alarm (indicated by a red fault icon on the virtual device). Secondly, we implement a data caching strategy. The edge gateway features a built-in cache with a capacity of 1GB, which stores data during network interruptions. Once the connection is restored, the stored data will be resent in chronological order to prevent data loss.

**6 Virtual-Real mapping in the workshop**

Virtual-real mapping serves as the cornerstone of digital twins.By establishing a multi-dimensional connection between virtual models and physical entities, it facilitates a closed loop of "physical world occurrence → data transmission → virtual world reflection → decision optimization → physical world execution." The virtual-real mapping of the workshop's production line is categorized into four levels（shown in Table 3）:

**Table 3.Virtual real mapping hierarchy table of base production line**

| **Mapping hierarchy** | **Core content** | **Technical objectives** |
| --- | --- | --- |
| Geometric mapping | The virtual model corresponds to the shape, size and position of the physical entity | Geometric error ≤ 0.1mm, position deviation ≤ 1mm |
| Behavior mapping | The motion trajectory and action logic of the virtual model are consistent with the physical equipment | Action synchronization error ≤ 50ms |
| State mapping | The virtual model reflects the operation status of physical equipment in real time (such as operation/fault) | Status update delay ≤ 100ms |
| Parameter mapping | The process parameters (such as temperature and pressure) of the virtual model are synchronized with the physical equipment in real time | Parameter error ≤ 1% |

**6.1 Implementation of Geometric Mapping**

**6.1.1 Coordinate System Unification**

Firstly, establish a global coordinate system for the workshop. The center of the injection molding machine nozzle is designated as the origin (0,0,0), with the X-axis running along the conveyor belt, the Y-axis perpendicular to it, and the Z-axis pointing upwards. Both physical equipment and virtual models are positioned based on this coordinate system. A laser tracker (with an accuracy of ±0.05mm) is utilized to calibrate the coordinates of the physical equipment, ensuring alignment with the virtual models. Secondly, consider dynamic geometric adjustments. For components prone to deformation, such as robotic arms, the "Finite Element Analysis" module in NX is employed to calculate stress-induced deformation. Real-time load data from the physical equipment is obtained via OPC UA, enabling dynamic adjustments to the geometry of the virtual model. For instance, when a robotic arm grasps a heavy object weighing 5kg, the virtual model automatically simulates a 0.5mm bending deformation based on the load signal from the physical sensor.

**6.1.2 behavior mapping implementation**

(1) Kinematic Mapping

The kinematic model of the manipulator is constructed using the D-H parameter method, and the mathematical relationship between the joint variables and the end effector pose is defined in the NX software. The joint angle data of the physical manipulator is obtained in real time through the OPC UA protocol and used to drive the rotation of the virtual manipulator's joints, ensuring that the virtual and actual motion trajectories are highly consistent, with a trajectory deviation controlled within 1 mm.

(2) Logical Behavior Mapping

The control logic of the virtual model is synchronized with the physical PLC program: The ladder diagram program of the physical PLC is imported into the virtual environment through the "PLC Open" interface provided by NX MCD, thereby realizing the reproduction of the control logic.For example, if the logical relationship "Injection molding completed → Conveyor belt starts" is set in the physical PLC, in the virtual model, it is achieved through a "signal trigger" mechanism. When the virtual injection molding machine sends a ejection signal of "1", the system will automatically trigger and start the virtual conveyor belt.

**6.1.3 Implementation of state and parameter mapping**

A real-time synchronization strategy is employed. Firstly, timestamp-based synchronization is utilized, where physical devices attach precise timestamps (with a precision of 1 ms) when collecting data. Upon receiving the data, the virtual model aligns states and parameters according to these timestamps to avoid asynchronous discrepancies. Secondly, event-triggered synchronization is implemented; when a physical device experiences a state change (such as a fault or shutdown), an event signal (e.g., "Fault=1") is immediately sent out. The virtual model updates its state instantaneously upon receiving this signal (for instance, displaying a shutdown animation for the virtual device).

A deviation correction mechanism is adopted. Regular calibration is conducted every eight hours using laser interferometry to measure the actual position of the physical robotic arm and compare it with that of the virtual model. Deviations are calculated, generating correction parameters (e.g., X-axis compensation +0.02 mm), which are then written into the virtual model via OPC UA protocol. Additionally, dynamic compensation techniques are applied; specifically addressing the lagging characteristics of temperature in injection molding machine barrels, a temperature prediction model based on BP neural networks has been established within the virtual model. This model utilizes real-time temperature data from physical sources along with historical curves to proactively adjust virtual temperature values, ensuring that deviations between both remain within ±1°C.

**6.2 Application scenarios based on mapping**

**6.2.1 Real-time monitoring and visualization**

In the NX virtual environment, a digital dashboard for the production line is constructed to display the operational status of each device in real time (green = running, yellow = standby, red = fault), along with output data (hourly base production) and quality metrics (pass rate). Through virtual-physical mapping, the internal states of devices are visualized. internal conditions that cannot be directly observed in physical equipment (such as the screw position of an injection molding machine) are intuitively presented through the transparency feature of virtual models, assisting operators in assessing equipment status.

**6.2.2 Virtual debugging and process optimization**

Before launching a new production line, a full-process simulation is conducted within a virtual model. Parameters for virtual debugging (such as robotic arm assembly speed) are transmitted to physical devices via OPC UA to validate process feasibility. To address quality fluctuations during base assembly, adjustments are made to machine vision positioning parameters (e.g., template matching threshold) within the virtual model. The assembly deviations under different parameter settings are simulated, allowing optimal parameters to be applied back into the physical system through virtual-physical mapping—resulting in an increase in pass rate from 95% to 99.5%.

**6.2.3 Fault Diagnosis and Prediction**

When physical equipment experiences faults (e.g., robotic arm jamming), the virtual model reproduces fault phenomena through state mapping while simulating fault evolution processes using historical data (such as current changes prior to jamming) within a virtual environment—thereby identifying root causes of failures (e.g., bearing wear).

Based on parameter trend analysis derived from virtual-physical mapping, A long-term record of clamping force variation curves for injection molding machines is maintained by the virtual model; when these curves exhibit a slope exceeding predetermined thresholds (for instance, a monthly decline greater than 2%), it predicts mold wear and generates maintenance alerts proactively.

**7 Case application and verification**

**7.1 Application scenarios**

Currently, the production of smoke alarms at an electronic technology company primarily relies on manual labor. The outdated method of integrating workshop production data, coupled with a lack of effective monitoring tools, results in low transparency and difficulty in managing workshop operations, significantly hindering production efficiency. To address these issues, the company is advancing the intelligent upgrading and transformation of its workshops, with the goal of enhancing the intelligence of production lines, optimizing workshop management, and increasing workshop transparency. The digital twin workshop architecture and four key technologies presented in this paper offer an effective solution for the smoke alarm base production line.

By utilizing the twin model modeling approach, data acquisition and transmission solutions outlined in this paper, along with the process flow of the actual production line,we have developed a digital twin model for the smoke alarm base production line and established a virtual workshop. Powered by real-time data, the virtual workshop precisely mirrors the production and processing activities in the physical workshop.Building on this foundation,we have deployed and implemented a three-dimensional, visually enhanced real-time monitoring system in the workshop, leveraging digital twin technology, to enable real-time tracking of production status and assist staff in comprehensively understanding the workshop's production status and operational information. Figure8 displays the main interface of the twin system.

There are four main buttons at the top of the main interface, i.e. start, reset, pause and stop, which can manually control the start, pause, stop, reset and other functions of the production line. There is a current alarm display column under the four buttons. If the production line fails, it will scroll through the interface. The real-time output is displayed on the left side of the middle position of the main interface in real time. The simulation operation module can simulate key unit operations such as injection molding, assembly, packaging, and reclaiming before actual production. The right reset initialization status module manually initializes the main nodes of the production line process. At the bottom of the main interface, there are five button modules: manual page, alarm page, parameter page, debugging page and main page. The manual page can realize the function of manually operating the production line, the parameter page can display the production parameters of each equipment of the production line in real time, and the debugging page can debug each unit of the production line.

**Figure8 Twin system main interface**

Figure 9 shows the workshop monitoring interface. The middle position of the interface displays the working data of the production line in real time, and the corresponding equipment position displays the working status of the equipment in real time. Green indicates normal operation, yellow indicates standby, and red indicates fault alarm. The upper left corner of the interface displays the humidity, temperature, startup rate, gas supply pressure, utilization rate and other data of the workshop in real time. The upper right corner can display the alarm information of the production line in real time. An emergency stop button is set at the lower part of the interface, which can be manually stopped in case of emergency. The machine vision detection information that cannot be seen in the model is displayed at the bottom of the interface in real time. At the bottom right of the interface is the workshop data summary chart, which can display the workshop running time, product qualification rate summary, workshop output and other data in real time.

**Figure 9 System monitoring interface**

After meticulous on-site debugging and practical implementation,the system has demonstrated exceptional performance.It operates extremely smoothly, maintaining a stable average response time of 48.6ms (±3.2ms), fulfilling the real-time demands for system data acquisition and transmission. Currently, a synchronous and efficient operational state has been largely achieved between the twin (virtual) workshop and the physical workshop.

The digital twin system primarily fulfills three key functions. In terms of tracking production progress, the system is capable of precisely depicting the real-time advancements of each stage in the smoke alarm base production line, spanning from raw material input to finished product output. The completion status and time nodes of each step are clearly discernible, enabling staff to promptly identify production bottlenecks, agilely adjust resource allocation, and guarantee a seamless and unimpeded production flow.

In terms of equipment status monitoring, the system provides comprehensive surveillance of all types of equipment on the production line, real-time collecting operational parameters such as temperature, rotational speed, and vibration. Upon detecting any abnormal conditions in the equipment, the system promptly issues early warnings to alert maintenance personnel to potential failure risks in advance, enabling them to arrange for preventive maintenance. This effectively minimizes the equipment failure rate and mitigates production disruptions caused by equipment shutdowns.

For quality control, by conducting an in-depth analysis of production process data and correlating it with product quality data, we can precisely pinpoint the stages and causes of quality issues. For instance, if welding defects are found in a batch of smoke alarm bases, the system can swiftly trace these back to welding equipment parameters, operators, and environmental factors at the time, furnishing a solid foundation for quality enhancement and aiding enterprises in elevating their product quality.

In addition, the system boasts energy management capabilities, enabling real-time monitoring of energy consumption across the workshop and analysis of energy efficiency for each piece of equipment and production stage. Leveraging intelligent algorithms, it offers energy optimization recommendations for workshops, assisting enterprises in reducing energy consumption and achieving green production. Thanks to these functions, the digital twin monitoring system significantly enhances the intelligence level and management efficiency of the smoke alarm base production line, thereby generating greater value for enterprises.Figure8 depicts the system monitoring interface.

**7.2 Operation effect**

(1) Twin model accuracy verification

Geometric mapping accuracy: randomly select 10 base parts and measure the key dimensions (such as diameter and height) of the physical parts and the virtual model, with an average error of 0.08mm, meeting the design requirements (≤ 0.1mm).

Behavior synchronization accuracy: record the physical execution time and virtual simulation time of the manipulator grasp action, and the synchronization error is 35ms, which is lower than the threshold (50ms).

(2) Productivity improvement

Virtual debugging cycle: traditional physical debugging takes 72 hours, while virtual debugging based on digital twin takes only 24 hours, with an efficiency increase of 66.7%;

Equipment utilization rate: through real-time monitoring and scheduling optimization of virtual and real mapping, the equipment utilization rate increased from 85% to 92%, and the daily output increased to 5300 pieces.

(3) Quality and cost optimization

Quality inspection: the machine vision system improved the accuracy of defect recognition from 96% to 99.2% by optimizing the parameters of the virtual model;

Energy consumption reduction: optimization of injection molding process parameters based on virtual model (e.g. pressure holding time is shortened from 3 seconds to 2.5 seconds), and energy consumption per unit product is reduced by 8%.

**6.Concern of the reviewer:**

**Transitions between sections are weak. For instance, the transition from model construction (Section 3.1) to information system design (Section 3.2) lacks connective narrative. The authors should add brief transitional paragraphs to clarify how each section builds on the previous one, thereby improving the overall coherence of the manuscript.**

**Our response:** We are grateful to the Reviewer for his elaborate work to find out our weakness.We readjusted the content of the chapter and added Chapter 5 and Chapter 6. Chapter 5 describes in detail how to build an information model for physical entities, collect physical entity data and transmit data through the information model. Chapter 6 describes how to realize the mapping between virtual model and physical entity.

**Revised text:**

By utilizing the twin model modeling approach, data acquisition and transmission solutions outlined in this paper, along with the process flow of the actual production line,we have developed a digital twin model for the smoke alarm base production line and established a virtual workshop. Powered by real-time data, the virtual workshop precisely mirrors the production and processing activities in the physical workshop.Building on this foundation,we have deployed and implemented a three-dimensional, visually enhanced real-time monitoring system in the workshop, leveraging digital twin technology, to enable real-time tracking of production status and assist staff in comprehensively understanding the workshop's production status and operational information. Figure8 displays the main interface of the twin system.

There are four main buttons at the top of the main interface, i.e. start, reset, pause and stop, which can manually control the start, pause, stop, reset and other functions of the production line. There is a current alarm display column under the four buttons. If the production line fails, it will scroll through the interface. The real-time output is displayed on the left side of the middle position of the main interface in real time. The simulation operation module can simulate key unit operations such as injection molding, assembly, packaging, and reclaiming before actual production. The right reset initialization status module manually initializes the main nodes of the production line process. At the bottom of the main interface, there are five button modules: manual page, alarm page, parameter page, debugging page and main page. The manual page can realize the function of manually operating the production line, the parameter page can display the production parameters of each equipment of the production line in real time, and the debugging page can debug each unit of the production line.

**Figure8 Twin system main interface**

Figure 9 shows the workshop monitoring interface. The middle position of the interface displays the working data of the production line in real time, and the corresponding equipment position displays the working status of the equipment in real time. Green indicates normal operation, yellow indicates standby, and red indicates fault alarm. The upper left corner of the interface displays the humidity, temperature, startup rate, gas supply pressure, utilization rate and other data of the workshop in real time. The upper right corner can display the alarm information of the production line in real time. An emergency stop button is set at the lower part of the interface, which can be manually stopped in case of emergency. The machine vision detection information that cannot be seen in the model is displayed at the bottom of the interface in real time. At the bottom right of the interface is the workshop data summary chart, which can display the workshop running time, product qualification rate summary, workshop output and other data in real time.

**Figure 9 System monitoring interface**

6. **Concern of the reviewer:**

**The figures provided in the article are mostly conceptual, focusing on architecture and models, with a lack of actual data and real-world scenarios.**

**Our response:** We are grateful to the Reviewer for his elaborate work to find out our weakness.

We added Chapter 3 to describe the real scene, added chapter 4.4 to describe how to carry out workshop modeling in NX, added Chapter 5 to describe how to carry out data collection and data transmission between the model and physical entities, and added Chapter 7 to describe the application scenario in detail, and compared the digital twin technology with traditional production.

**Revised text:**

**5 Data acquisition and transmission**

**5.1 Workshop information system architecture**

The core of digital twin is modeling and data interaction. The design of a reasonable digital twin workshop information physical fusion system is the basis for the construction of virtual real interaction in digital workshop, and also the key to solve the problem of information island in digital workshop.In the production workshop for smoke alarm bases, there are numerous field devices, each with its unique protocol.To address this, a unified data model is necessary to facilitate data transmission and retrieval.The workshop has integrated OPC UA servers into each intelligent device on the production line. By utilizing information modules to analyze data collected from the underlying devices, these devices are connected to nodes within the OPC UA server's address space. Interaction for big data analysis is achieved through the connection between OPC UA server endpoints.The OPC UA information network structure comprises three layers: the equipment layer, the communication layer, and the information layer. The underlying equipment primarily includes production equipment, auxiliary devices, and sensors. At the equipment layer, operational and status data generated by the normal operation of the equipment are first encapsulated in the OPC UA server. Subsequently, this data is transmitted to higher-level applications using OPC UA's subscription/publish or query modes. Devices can directly communicate with the OPC UA server through OPC UA clients. The communication layer serves as the bridge between underlying facilities and information systems, as well as external networks and cloud applications. At the information layer, the OPC UA client is used to obtain various data from the equipment layer and store it in a local database for use by resource planning (EPR) and manufacturing execution systems (MES). Figure6 illustrates the workshop's information system architecture.

**Figure6 The workshop's information system architecture**

**5.2 Data acquisition system design**

**5.2.1 Types and parameters of collected data**

The data to be collected by the substructure production line is divided into four categories, and the specific parameters are as follows（shown in Table 1）:

**Table 1. Data acquisition parameters**

| **Data type** | **Collection object** | **Key parameter** | **Acquisition frequency** | **Accuracy requirements** |
| --- | --- | --- | --- | --- |
| Equipment status data | Injection molding machine, manipulator, etc | Operation status (operation/shutdown/fault), motor speed, temperature | 100ms | State recognition accuracy 100% |
| Process parameter data | Injection molding machine | Injection pressure, holding time, mold temperature | 50ms | ±0.1MPa（pressure） |
| Quality inspection data | Machine vision system | Positioning deviation, defect type, dimension measurement value | 1s/time (per product) | ±0.01mm（size） |
| Logistics information data | Conveyor belt , sealing machine | Base position, conveying speed, packing quantity | 200ms | position error ±5mm |

**5.2.2 Hardware acquisition architecture**

Sensor layer:

Various sensors are installed on the physical equipment: pressure sensor (range 0-200mpa) and temperature sensor (PT100, -50~200 ℃) are installed on the injection molding machine.

The encoder (resolution 1024 lines) installed on the conveyor belt is used for speed detection, and the laser displacement sensor (accuracy ± 0.001mm) is installed at the assembly station to detect the height of the insert.

Edge layer:

The industrial gateway (such as Siemens IOT 2050) is used as the data acquisition node, and the PLC is connected through PROFINET bus to obtain the equipment control signal.

OPC UA server (such as kepserverex) is built in the gateway to convert sensor and PLC data into OPC UA protocol format.

Transport layer:

Industrial Ethernet (Gigabit bandwidth) is used to build the communication network. The equipment and gateway are connected by wire (tcp/ip protocol), and wireless backup (5g) is supported between the gateway and the server.

The network delay is controlled within 50ms, and the "priority queue" ensures the priority transmission of key data (such as fault signals).

**5.3 OPC UA information model construction**

**5.3.1 Information model construction process**

The OPC UA based information model needs to map the physical entity and data relationship of the base production line. The specific construction steps are as follows:

(1) Namespace definition

Define custom namespace (URI: http://base-production-line.com/opcua ）, distinguished from OPC UA standard namespace, Namespace contains object nodes such as "production line", "production unit" and "assembly unit".

(2) Object level design

Root node: baseproductionline.

First level child nodes: injectionunit (production unit), assemblyunit (assembly unit), inspectionunit (inspection unit), packingunit (packaging unit).

Secondary child nodes: each unit contains specific equipment objects (such as injectionmoldingmachine, robot, camera, etc.).

Three level child nodes: the device object contains variables and method nodes such as status, parameters, and operation (such as the injectionpressure variable and the start method).

(3) Variable node attribute definition

Take the "injection pressure" variable of the injection molding machine as an example, define the attributes（shown in Table 2）:

**Table 2. Variable node attribute definition**

| **Data type** | Float |
| --- | --- |
| **Engineering unit** | MPa |
| **Minimum value** | 0 |
| **Maximum value** | 100 |
| **Access rights** | Readable and writable (support the virtual model to adjust the parameters of physical devices |
| **Sampling interval** | 50ms |

**5.3.2 Information Model for the Base Production Line**

On the organizational structure, functional modules, and data flow requirements among these modules in the smoke alarm base production workshop, the physical equipment and production process data within the workshop are digitally described and modeled, organized according to information modeling principles to form the digital workshop information model for smoke alarm base production. This model encompasses a static attribute set, a process attribute set, and a functional component set. The workshop information model aligns with the entire workshop, the functional component set corresponds to each functional module within the workshop, and the resource component set represents the various resources involved in each module. The attribute set signifies a collection of attributes, which serve as the fundamental unit of description in the information model. With a fixed structure, attributes are used to delineate the associations among devices, components, attribute sets, and attributes themselves.

The production line for smoke alarm bases boasts a variety of equipment types, posing significant integration challenges. The integration of this equipment and the acquisition of data are crucial to the performance of digital workshops.The OPC Unified Architecture(OPC UA) offers a standardized communication mechanism that can be synchronous, asynchronous, or distributed. This mechanism allows for the access of different types of data both horizontally and vertically. Based on OPC UA, this paper constructs an information model for the digital twin workshop of the smoke alarm base production line, as illustrated in Figure 7. The model comprises a static attribute set, a process attribute set, and a functional component set. The static attribute set corresponds to the fundamental static information of workshop operations, encompassing basic workshop information (such as the workshop's name, location, area, and person in charge), production organization (such as the departments and workgroups within the workshop), the work calendar (such as working days and rest days), and production work orders (order information obtained by the MES system from the ERP system) [14]. The process attribute set corresponds to the dynamic data of workshop processes, typically comprising aggregated statistical data from various aspects of the workshop and order tracking information, which is used to provide feedback to the ERP system. This includes order tracking, production statistics, quality statistics, inventory statistics, and maintenance statistics[15].The functional component set aligns with various production type modules within the production workshop, encompassing units for production, assembly, quality inspection, packaging, among others. Each production type module comprises a static attribute set, a procedural attribute set, and a resource component set. The static attribute set denotes the fundamental information pertinent to each module, including work plans, production techniques, and shift outputs. The procedural attribute set represents the production process information for each resource module, encompassing production plan tracking, operational data, and production statistics. The resource component set signifies the diverse resources associated with each module, such as injection molding machines, industrial robots, and quality inspection equipment.

**Figure7 Information model of digital production workshop for smoke alarm base production line**

**5.4 Data transmission realization**

The integration of NX and OPC UA serves as the pivotal aspect for enabling digital twin virtual-to-real data interaction within the base production line. By configuring an OPC UA client within the NX environment, establishing mechanisms for data subscription and publication, and devising strategies for handling exceptions, a real-time data pathway between virtual models and physical entities is established. This integration solution must fulfill the criteria of industrial-grade real-time performance (with a data update delay of ≤100ms), security (preventing data leakage and tampering), and reliability (ensuring no single point of failure). The detailed implementation process is outlined as follows:

(1) OPC UA client configuration in NX

First, enable the "OPC UA client" module in the NX MCD, connect the OPC UA server of the edge gateway through the "server address", then configure the security policy, use the "basic256sha256" encryption algorithm, and import the server certificate to complete the identity authentication.

(2) Data subscription and publishing mechanism

First, the virtual model subscribes to the physical device data.Associates variables (such as the "injection pressure" of the virtual injection molding machine) in NX with the corresponding node of OPC UA server (ns=1; s=injectionunit.injectionmoldingmachine.injectionpressure), and sets the subscription cycle of 50ms. Secondly, the virtual model issues control instructions. When adjusting parameters (such as manipulator speed) through virtual debugging, the NX client publishes the new parameter values to the "robot.speed" node of the OPC UA server, and the physical PLC acquires and executes the instructions by subscribing to this node.

(4) Data Exception Handling

Firstly, we adopt the "heartbeat detection" mechanism. If no data is received from the physical device within 100 milliseconds, NX automatically labels the data as "invalid" and triggers an alarm (indicated by a red fault icon on the virtual device). Secondly, we implement a data caching strategy. The edge gateway features a built-in cache with a capacity of 1GB, which stores data during network interruptions. Once the connection is restored, the stored data will be resent in chronological order to prevent data loss.

**6 Virtual-Real mapping in the workshop**

Virtual-real mapping serves as the cornerstone of digital twins.By establishing a multi-dimensional connection between virtual models and physical entities, it facilitates a closed loop of "physical world occurrence → data transmission → virtual world reflection → decision optimization → physical world execution." The virtual-real mapping of the workshop's production line is categorized into four levels（shown in Table 3）:

**Table 3.Virtual real mapping hierarchy table of base production line**

| **Mapping hierarchy** | **Core content** | **Technical objectives** |
| --- | --- | --- |
| Geometric mapping | The virtual model corresponds to the shape, size and position of the physical entity | Geometric error ≤ 0.1mm, position deviation ≤ 1mm |
| Behavior mapping | The motion trajectory and action logic of the virtual model are consistent with the physical equipment | Action synchronization error ≤ 50ms |
| State mapping | The virtual model reflects the operation status of physical equipment in real time (such as operation/fault) | Status update delay ≤ 100ms |
| Parameter mapping | The process parameters (such as temperature and pressure) of the virtual model are synchronized with the physical equipment in real time | Parameter error ≤ 1% |

**6.1 Implementation of Geometric Mapping**

**6.1.1 Coordinate System Unification**

Firstly, establish a global coordinate system for the workshop. The center of the injection molding machine nozzle is designated as the origin (0,0,0), with the X-axis running along the conveyor belt, the Y-axis perpendicular to it, and the Z-axis pointing upwards. Both physical equipment and virtual models are positioned based on this coordinate system. A laser tracker (with an accuracy of ±0.05mm) is utilized to calibrate the coordinates of the physical equipment, ensuring alignment with the virtual models. Secondly, consider dynamic geometric adjustments. For components prone to deformation, such as robotic arms, the "Finite Element Analysis" module in NX is employed to calculate stress-induced deformation. Real-time load data from the physical equipment is obtained via OPC UA, enabling dynamic adjustments to the geometry of the virtual model. For instance, when a robotic arm grasps a heavy object weighing 5kg, the virtual model automatically simulates a 0.5mm bending deformation based on the load signal from the physical sensor.

**6.1.2 behavior mapping implementation**

(1) Kinematic Mapping

The kinematic model of the manipulator is constructed using the D-H parameter method, and the mathematical relationship between the joint variables and the end effector pose is defined in the NX software. The joint angle data of the physical manipulator is obtained in real time through the OPC UA protocol and used to drive the rotation of the virtual manipulator's joints, ensuring that the virtual and actual motion trajectories are highly consistent, with a trajectory deviation controlled within 1 mm.

(3) Logical Behavior Mapping

The control logic of the virtual model is synchronized with the physical PLC program: The ladder diagram program of the physical PLC is imported into the virtual environment through the "PLC Open" interface provided by NX MCD, thereby realizing the reproduction of the control logic.For example, if the logical relationship "Injection molding completed → Conveyor belt starts" is set in the physical PLC, in the virtual model, it is achieved through a "signal trigger" mechanism. When the virtual injection molding machine sends a ejection signal of "1", the system will automatically trigger and start the virtual conveyor belt.

**6.1.3 Implementation of state and parameter mapping**

A real-time synchronization strategy is employed. Firstly, timestamp-based synchronization is utilized, where physical devices attach precise timestamps (with a precision of 1 ms) when collecting data. Upon receiving the data, the virtual model aligns states and parameters according to these timestamps to avoid asynchronous discrepancies. Secondly, event-triggered synchronization is implemented; when a physical device experiences a state change (such as a fault or shutdown), an event signal (e.g., "Fault=1") is immediately sent out. The virtual model updates its state instantaneously upon receiving this signal (for instance, displaying a shutdown animation for the virtual device).

A deviation correction mechanism is adopted. Regular calibration is conducted every eight hours using laser interferometry to measure the actual position of the physical robotic arm and compare it with that of the virtual model. Deviations are calculated, generating correction parameters (e.g., X-axis compensation +0.02 mm), which are then written into the virtual model via OPC UA protocol. Additionally, dynamic compensation techniques are applied; specifically addressing the lagging characteristics of temperature in injection molding machine barrels, a temperature prediction model based on BP neural networks has been established within the virtual model. This model utilizes real-time temperature data from physical sources along with historical curves to proactively adjust virtual temperature values, ensuring that deviations between both remain within ±1°C.

**6.2 Application scenarios based on mapping**

**6.2.1 Real-time monitoring and visualization**

In the NX virtual environment, a digital dashboard for the production line is constructed to display the operational status of each device in real time (green = running, yellow = standby, red = fault), along with output data (hourly base production) and quality metrics (pass rate). Through virtual-physical mapping, the internal states of devices are visualized. internal conditions that cannot be directly observed in physical equipment (such as the screw position of an injection molding machine) are intuitively presented through the transparency feature of virtual models, assisting operators in assessing equipment status.

**6.2.2 Virtual debugging and process optimization**

Before launching a new production line, a full-process simulation is conducted within a virtual model. Parameters for virtual debugging (such as robotic arm assembly speed) are transmitted to physical devices via OPC UA to validate process feasibility. To address quality fluctuations during base assembly, adjustments are made to machine vision positioning parameters (e.g., template matching threshold) within the virtual model. The assembly deviations under different parameter settings are simulated, allowing optimal parameters to be applied back into the physical system through virtual-physical mapping—resulting in an increase in pass rate from 95% to 99.5%.

**6.2.3 Fault Diagnosis and Prediction**

When physical equipment experiences faults (e.g., robotic arm jamming), the virtual model reproduces fault phenomena through state mapping while simulating fault evolution processes using historical data (such as current changes prior to jamming) within a virtual environment—thereby identifying root causes of failures (e.g., bearing wear).

Based on parameter trend analysis derived from virtual-physical mapping, A long-term record of clamping force variation curves for injection molding machines is maintained by the virtual model; when these curves exhibit a slope exceeding predetermined thresholds (for instance, a monthly decline greater than 2%), it predicts mold wear and generates maintenance alerts proactively.
