# Supplementary material for: Development and implementation of a Digital Twin workshop for a smoke alarm production line
Source: PLoS One. 2026 Jan 29;21(1):e0341224. doi: 10.1371/journal.pone.0341224 (PMC12854460; doi:10.1371/journal.pone.0341224)
Supplement: Attachment — Submitted filename: Response to the Reviewers comments2.docx [file pone.0341224.s003.docx]

**Dear Editor and Reviewers,**

We thank you very much for giving us another chance to revise and improve our manuscript. We try our best to revise the paper according to the reviewers’ suggestions, and for those comments we have different opinions, we have explained in detail. A list of changes and responses are listed below.

We hope the revised paper has addressed all the reviewers’ concerns and reached the requirements of the journal for publishing.

Thank you very much!

The authors

**Responses to Reviewers’ Comments**

**Reply to reviewer #1**

1. **Concern of the reviewer:**

**The innovations are rather engineering-oriented and lack theoretical depth. For example, while the construction of the digital twin model is detailed, it does not introduce novel algorithms (such as AI-driven predictive models) or cross-innovations with other fields (such as integration with edge computing or blockchain). The authors are advised to emphasize the differences with existing research, for example, through comparative analysis, highlighting the unique advantages of this approach (such as the efficient mapping of multi-source heterogeneous data).**

**Our response:** We are grateful to the Reviewer for his elaborate work to find out our weakness.We have enhanced the theoretical depth in Sections 4, 5, and 6.Section 4 elaborates on how edge computing and AI prediction methods are applied to construct the digital twin model.
Section 5 expands on the application of edge and AI algorithms in data acquisition and transmission.Section 6 provides a detailed explanation of implementing workshop-level virtual-physical mapping using the multi-scale dynamic mapping error compensation theoretical model.
Due to the extensive content, these details are not fully listed here. We kindly invite the reviewers to examine the complete descriptions in the manuscript.

2. **Concern of the reviewer:**

**There are minor grammatical errors and awkward phrasing, such as the use of "compared with transmission production" in the Abstract (a possible typo for "traditional production"). The reference to "untraceable quality issues" in the Introduction could be more precisely replaced with "untraceable quality defects."**

**Our response:** Thanks very much to the Reviewer for his meticulous work to find out our mistake , We have corrected the wrong content in the article. Due to the large number of corrected content, the red part is marked in the manuscript,we kindly request that the reviewers evaluate these changes directly in the manuscript.

3. **Concern of the reviewer:**

**Some of the results (such as an 8% decrease in energy consumption and a 3.2% increase in defective product identification rate) lack systematic comparison with existing research, making it difficult to highlight their advantages.**

**Our response:** We are grateful to the Reviewer for his elaborate work to find out our weakness.We have rewritten section 7.2 and compared the existing technology with traditional production in detail.

**Revised text:**

**7.2 Operational performance comparison**

The traditional production process is as follows: The injection molding machine first produces the base component of the smoke alarm, followed by manual preliminary screening and transportation to the assembly stage. On the assembly line, workers assemble several metal parts with the base, after which another round of manual screening is conducted. Subsequent steps include manual folding of cartons, placing the assembled products into the cartons, folding the outer boxes, manual plastic sealing, packing, and palletizing. The entire process requires 30 operators to meet production capacity demands.

The existing production line has undergone automation transformation, significantly optimizing the processes: After injection molding, the base parts are automatically unloaded by the injection molding machine’s robotic arm and transported via conveyor belt to an integrated machine for pressing metal parts. A robot then grasps the product for visual inspection. After the metal parts are pressed, a secondary visual inspection is conducted. Qualified products are then transported to an automatic boxing system—a carton opening machine automatically unfolds and positions the cartons, and a robot places the products inside. The products are then sequentially sent via conveyor belt to a sealing machine for initial and secondary sealing, followed by automatic edge sealing by a sealing machine, automatic packing by a packing machine, and finally automatic palletizing. The entire production line requires only one operator to manage auxiliary material replenishment.

It is precisely based on this automation foundation that modern digital twin technology has been deeply integrated, achieving a leap from "machines replacing manual labor" to "data-driven intelligence," resulting in significant differences across multiple dimensions compared to traditional production models.

Comparative analysis: Traditional Production Model (Labor-Intensive) vs. Modern Digital Twin Production Line.

Traditional Production Model (Labor-Intensive)
In the traditional production model, the entire manufacturing process of smoke alarms heavily relies on manual operations. Its core characteristics include high labor input, low efficiency, high variable costs, and process opacity.

Production Efficiency: As over 15 processes primarily depend on manual work, including screening, assembly, packaging, and handling, the production line’s cycle time is severely constrained by workers’ skill levels and fatigue. The overall production cycle for a single product is lengthy, with obvious bottlenecks in capacity improvement and a low daily production ceiling.

Labor Costs: To meet basic production capacity requirements, the production line must employ at least 30 operators. Assuming a comprehensive labor cost (including salaries, social security, management, etc.) of approximately 6,000 RMB per person per month, the monthly direct labor cost alone reaches 180,000 RMB. This not only represents a significant ongoing expense but also comes with challenges in recruitment, training, and management.

Quality Costs: Quality inspection relies entirely on manual visual screening, which has relatively high rates of missed and false detection, resulting in a consistently high product defect rate (2%-5%). This leads to high rework costs, potential material waste, and customer complaint risks. When quality issues occur, it is difficult to trace the root cause.

Overall Costs: Although the initial equipment investment for a traditional production line is low, its long-term operational variable costs are extremely high. Soaring and continuously rising labor costs consistently erode profit margins, and scaling production almost linearly increases labor requirements, demonstrating clear diseconomies of scale. The production process operates like a "black box," with management decisions based on experience rather than data support.

Modern Digital Twin Production Line (Technology-Driven + Data Intelligence)
Through comprehensive automation upgrades and the integration of digital twin technology, the new production line achieves unmanned intelligent production and end-to-end digital management. Its core characteristics include low labor input, high efficiency, high quality, high predictability, and full process transparency.

Production Efficiency: Automated equipment enables 24/7 continuous production, with fixed equipment cycle times far exceeding manual operations. The digital twin system, through real-time data collection and virtual simulation, continuously optimizes the production process, improving production efficiency by 400% to 600% compared to the traditional model. Virtual commissioning technology reduces product changeover time by over 50%, significantly enhancing production flexibility.

Labor Costs: The entire complex process requires only one operator to manage auxiliary material replenishment and system monitoring. Monthly direct labor costs plummet to approximately 6,000 RMB, saving 174,000 RMB per month compared to the traditional model—a reduction of 96.7%. The role of employees shifts from operators to technical supervisors.

Quality Costs: Robotic vision systems conduct two rounds of 100% accurate and omission-free inspections, combined with full-process parameter monitoring (e.g., injection molding temperature, pressing force) enabled by digital twin technology, ensuring end-to-end traceability of product quality. The product defect rate drops significantly to below 0.1%, and quality issues can be immediately traced to their root causes, enabling predictive quality control.

Added Value of Digital Twin:

Real-Time Monitoring and Prediction: The virtual model mirrors the physical production line in real time, enabling real-time monitoring of equipment status and fault prediction.

Predictive Maintenance: Data analysis predicts equipment maintenance needs, reducing unplanned downtime by over 70%.

Process Optimization: Simulation tests different parameter combinations to continuously optimize production processes, improving OEE (Overall Equipment Efficiency) by 5%-15%.

Remote Management: Supports remote monitoring and decision-making, significantly enhancing management efficiency and quality control capabilities.

Overall Costs and Return on Investment: Although the initial investment for a digital twin production line is higher (including sensors, IoT devices, simulation software, etc.), it typically recovers the investment within 2-4 years by drastically reducing labor costs, quality costs, and downtime losses. Thereafter, it generates significant profit gains and offers stronger scalability.

From a quantitative perspective, the automation transformation empowered by digital twin technology brings fundamental changes: Replacing 30 workers with 1 operator, achieving multiples of efficiency improvement, and orders-of-magnitude quality enhancement. More importantly, digital twin technology transforms the production process from a "black box" to "transparent," from "experience-driven" to "data-driven," and from "reactive" to "predictive and proactive." This shift from "labor-intensive" to "technology-intensive + data-intelligent" not only significantly reduces operational costs but also greatly enhances quality control capabilities, production flexibility, and decision-making precision. It serves as an exemplary path for the transformation and upgrading of modern manufacturing.

4. **Concern of the reviewer:**

**The discussion section is descriptive and lacks analysis of the rationality and limitations of the results; the mechanism of energy consumption and yield improvement is not discussed in detail, only the results are given.**

**Our response:** We are grateful to the Reviewer for his elaborate work to find out our weakness.

We are compiled the conclusion and discussed it in detail from three aspects: the rationality of the results and the improvement mechanism, the limitations of the study, and the conclusion and outlook .

**Revised text:**

**8 Conclusion and Discussion**

As a critical bridge connecting the physical world and the virtual space, digital twin technology provides core support for the digital transformation of the manufacturing industry. To address the digitalization needs of a smoke alarm production line, this study constructed a hierarchical digital twin system encompassing "device-level, unit-level, and production line-level" layers. A multi-source heterogeneous data acquisition and transmission architecture based on OPC UA was designed and successfully implemented in a production line of an enterprise in Ningbo. Practical application demonstrated that the system operates stably and achieves core functions such as real-time production progress tracking, full-process quality control, and online equipment status monitoring. Compared with the traditional production mode, the production efficiency increased by 400%–600%, the product defect rate was significantly reduced to below 0.1%, and labor costs decreased by 96.7%. The following section will discuss the rationality of the results, improvement mechanisms, and research limitations.

(1) Analysis of result rationality and improvement mechanisms
The improvement in the defect recognition rate stems from the fundamental enhancement of traditional detection methods by the "virtual-real mapping" mechanism of the digital twin. Traditional production lines rely on manual sampling inspections and machine vision based on fixed thresholds, which are prone to issues such as missed detections (e.g., fine scratches) and misjudgments (e.g., grayscale deviations caused by lighting interference). This study achieved higher precision and robustness in quality control by establishing a virtual-real interactive defect recognition mechanism:

The virtual model utilized the NX Open API to call the OpenCV algorithm library, simulating the grayscale characteristics of various defects (e.g., pores and dimensional deviations) in the virtual environment, thereby forming a dynamically updated defect template library.

Images captured by the physical vision system were preprocessed by edge nodes and then compared and bidirectionally validated with the virtual template library in real time, effectively suppressing misjudgments caused by environmental interference.
 This mechanism increased the defect recognition rate from 96.3% to 99.5% (a net increase of 3.2%) while keeping the misjudgment rate below 0.1%, demonstrating the precision and reliability of the digital twin in quality control.

The reduction in energy consumption was primarily attributed to the digital twin's capability for dynamic optimization of equipment status and process parameters:

Equipment data (e.g., injection molding machine barrel temperature and robotic arm motor speed) were collected in real time via OPC UA, and an LSTM prediction model was employed to identify abnormal energy consumption. For instance, when the barrel temperature exceeded the process requirement by 2°C, the system automatically issued commands to adjust the heating power, saving approximately 1.2 kWh per hour for a single device.

The production line-level virtual model optimized logistics scheduling through path simulation and virtual commissioning, such as reducing the AGV transportation path by 15% to minimize no-load and ineffective energy consumption. Additionally, equipment operational status was dynamically adjusted based on order loads, avoiding energy waste caused by "overpowered equipment handling minor tasks."
 The above mechanisms collectively reduced the overall energy consumption of the production line by 8% and maintained stable energy efficiency even under order fluctuations (daily production volume variations of ±20%).

The improvement in production efficiency primarily relied on virtual commissioning replacing physical trial-and-error and dynamic scheduling through virtual-real collaboration:

Traditional production lines require shutdown for debugging during process changeovers (e.g., adjusting robot trajectories), which takes an average of 4–6 hours. This study pre-simulates the debugging process in a virtual environment to validate parameter feasibility in advance, reducing actual debugging time to within 30 minutes and shortening the production cycle per batch by 20%.

Bottleneck visualization is achieved through virtual-real mapping: the virtual interface displays the status of each workstation in real time (e.g., triggering an alert when assembly waiting time exceeds 5 minutes). The scheduling system dynamically optimizes material distribution based on this information, increasing the production line balance rate from 78% to 92%, and ultimately improving overall efficiency by 15.3%.

(2) Research Limitations
Although the system demonstrated significant results in practical applications, the following limitations remain.

Deviation in model accuracy under dynamic working conditions: The virtual model exhibited errors in geometric and behavioral mapping under high-load scenarios. For example, when the robotic arm grasped objects weighing over 5 kg, the actual arm bending deformation (approximately 0.8 mm) deviated from the finite element prediction (0.5 mm) by 0.3 mm. The primary cause was the virtual model's insufficient accounting for the reduction in elastic modulus due to accumulated material fatigue.

Bottleneck in edge node processing capability: When the current edge gateway (Siemens IoT 2050) processed high-frequency concurrent data from multiple devices (e.g., device status at 100 ms intervals and process parameters at 50 ms intervals), the data compression rate dropped from 90% to 75%. This resulted in transmission delays exceeding 100 ms for some non-critical data (e.g., environmental temperature and humidity), affecting the real-time synchronization efficiency of the virtual model.

The integration of blockchain introduces a cost-efficiency trade-off: while the consortium blockchain consensus mechanism (e.g., practical byzantine fault tolerance) ensures the immutability of critical data (e.g., quality inspection results, reducing the tampering rate to zero), it also introduces an approximate 20 ms data write latency and increases node maintenance costs by about 15% compared to conventional architectures. In small and medium-batch production scenarios, its economic viability remains to be optimized.

(3) Summary and Outlook
This study validated the feasibility of digital twin technology in the digital transformation of smoke alarm production lines. Its core value lies in breaking the "black box" of traditional manufacturing through a "virtual-real closed loop," achieving a fundamental shift from experience-driven to data-driven operations. Future work can be pursued in the following three aspects:

Introduce a material fatigue damage model to enhance prediction accuracy under high-load conditions.

Adopt an edge node cluster architecture to improve the processing capability for high-frequency concurrent data.

This study provides a reusable architectural and methodological framework for constructing digital twin systems in discrete manufacturing, though its generalizability and scalability require further validation in broader manufacturing scenarios, such as electronic assembly and automotive components.

5. **Concern of the reviewer:**

**Some paragraphs contain Chinglish, with lengthy sentences and insufficiently detailed diagram descriptions.**

**Our response:** We are grateful to the Reviewer for his elaborate work to find out our mistake. We have comprehensively revised the manuscript to address instances of Chinglish from the title through to the conclusion. The title has been changed from "Construction and Practice of Digital Twin Workshop for Smoke Alarm Base Production Line" to "Development and Implementation of a Digital Twin Workshop for a Smoke Alarm Production Line". Repetitive content between the introduction and the first chapter has been merged and refined. Long sentences have been restructured for improved clarity and flow, and figure and table descriptions have been expanded with greater detail. Among these revisions, the original Table 2 was deemed inappropriate and has been removed. Due to the extensive nature of the modifications, we kindly request that the reviewers evaluate these changes directly in the manuscript.
