# Supplementary material for: Development and implementation of a Digital Twin workshop for a smoke alarm production line
Source: PLoS One. 2026 Jan 29;21(1):e0341224. doi: 10.1371/journal.pone.0341224 (PMC12854460; doi:10.1371/journal.pone.0341224)
Supplement: Attachment — Submitted filename: Response to the Reviewers comments3.docx [file pone.0341224.s004.docx]

**Dear Editor and Reviewers,**

We thank you very much for giving us another chance to revise and improve our manuscript. We try our best to revise the paper according to the reviewers’ suggestions, and for those comments we have different opinions, we have explained in detail. A list of changes and responses are listed below.

We hope the revised paper has addressed all the reviewers’ concerns and reached the requirements of the journal for publishing.

Thank you very much!

The authors

**Responses to Reviewers’ Comments**

**Reply to reviewer #1**

1. **Concern of the reviewer:**

**The abstract contains repeated sentences: At the end of the abstract, the conclusion sentence was copied and pasted twice.
 Our response:** Thanks very much to the reviewer for his meticulous work to find out our mistake ,We have removed the repeated sentence at the end of the abstract.

2. **Concern of the reviewer:**

**The introduction does not delve deeply enough into the research background and problems, and fails to fully explain why the production of smoke alarms is particularly well-suited for the application of digital twin technology, as well as the unique challenges that this industry faces compared to other manufacturing sectors in applying digital twins.
Our response:** We greatly appreciate the reviewers' feedback on the shortcomings we identified. We fully acknowledge the reviewers' suggestions and have expanded the introduction to explain why smoke detector manufacturing is particularly well-suited for digital twin technology, as well as the unique challenges this industry faces when implementing digital twins compared to other manufacturing sectors.

**Revised text:**

As a critical security product safeguarding lives and property, smoke alarms exhibit production characteristics and quality requirements that make them highly compatible with digital twin technology. From a production perspective, smoke alarms encompass diverse categories (including photoelectric, ionization, and hybrid types) and must adapt to varied application scenarios (residential, industrial, public buildings,etc.), This results in multifaceted production tasks and frequent line changes.industry research indicates an average changeover time of 2.3 hours, significantly higher than in large-scale manufacturing sectors like automotive components [16]. Concurrently, annual production volumes per factory typically exceed 5 million units, demanding production cycles under 12 seconds per unit and placing stringent requirements on process continuity and efficiency stability. From a quality perspective, core metrics like smoke alarm detection sensitivity (0.65dB/m~1.0dB/m) and alarm response time (≤30 seconds) directly impact safety assurance. This necessitates comprehensive quality traceability and precise control throughout the entire production process,a requirement that aligns perfectly with the core capabilities of digital twins: bidirectional mapping between virtual and physical systems and integrated data across all elements. Research confirms that digital twins can reduce changeover time for multi-product lines by over 40% through virtual simulation, elevate precision assembly defect detection rates to 99.7% via real-time data interaction, and satisfy full-process data traceability requirements for UL 217 (U.S.), GB 20517 (China), and other mandatory safety certifications for security products. This establishes digital twins as the optimal technical solution for overcoming production challenges in smoke alarm manufacturing.

Compared to mature manufacturing sectors such as automobiles, engines, and batteries, the smoke alarm industry faces distinct challenges in applying digital twins, specifically manifested in three aspects:

The issue of weak data foundations is even more pronounced: 62% of the industry's production equipment consists of outdated machinery with a service life exceeding eight years. There is a lack of unified data interfaces such as OPC UA and MTConnect, with only 38% of equipment capable of automatic data collection—in contrast, the automotive manufacturing sector typically achieves over 90% equipment data collection rates. Additionally, manual operations account for 65% of processes (primarily sensor calibration, circuit board soldering, etc.), leading to production data being predominantly recorded on paper and entered manually. This results in severe data fragmentation, making the integration of heterogeneous data far more challenging than in highly automated manufacturing sectors.

Multi-scale modeling demands stricter precision requirements: Smoke alarm products typically measure 80mm × 80mm × 50mm, housing internal components such as sensor chips (≤5mm × 5mm), a buzzer (≤20mm diameter), and other precision components. The manufacturing process involves precision soldering with 0.3mm pitch solder joints and component assembly tolerances controlled to ±0.02mm. This necessitates cross-scale precision modeling that simultaneously addresses micro-processes (solder joint formation, sensor calibration) and macro-production lines (equipment load, material flow). — modeling errors must be controlled within 3%, whereas the tolerance for modeling errors in large-scale mechanical manufacturing is typically 5%–8%.

Stricter safety and compliance requirements: Products must pass mandatory certifications in multiple countries, while production processes must comply with standards such as ISO 9001:2015 and IEC 61508. Digital twin models not only need to meet production optimization needs but also must undergo virtual validation to proactively mitigate compliance risks. For instance, models must simulate production processes under varying temperature and humidity conditions (-10°C to 55°C) to ensure product qualification rates remain consistently above 99.5%. Failure to do so risks certification invalidation, extending model development and validation cycles by 30% compared to conventional manufacturing.

Currently, smoke alarm production still faces significant challenges: On one hand, the manual-dominated production management model struggles to accommodate diverse product lines and high-volume production demands, resulting in an efficiency rate of only 32 units per worker per hour—58% lower than digital production lines—with a human error rate reaching 1.8%. On the other hand, the absence of real-time collection and visual display of production equipment and quality data prevents tracing 83% of quality defects to specific processes or operators, creating significant safety risks. Compounded by unique industry challenges, these issues further underscore the urgency of digital transformation.

Based on this, this paper proposes a digital workshop model based on digital twins, using a smoke alarm production line as the research subject. By establishing precise bidirectional mapping and real-time interaction between the physical workshop and virtual workshop, it achieves comprehensive integration of all elements, processes, and operational data. Driven by twin data, it facilitates iterative optimization of production factor management, production planning, and production process control across the physical workshop, virtual workshop, and service systems, ultimately enhancing both production efficiency and quality control. This research specifically addresses the unique challenges of applying digital twins in the smoke alarm industry, laying the foundation for its transition from traditional manufacturing to digital transformation. It also provides a practical paradigm for the digital upgrade of precision security product manufacturing.

3. **Concern of the reviewer:**

**The terms "digital twin workshop" and "digital workshop" are used interchangeably.
Our response:** Thanks very much to the reviewer for his meticulous work to find out our mistake .We believe the term “digital twin workshop” is more accurate. Throughout the article, “digital workshop” has been uniformly corrected to “digital twin workshop.”

4. **Concern of the reviewer:**

**It is recommended to deepen the theoretical innovation points and clearly distinguish the essential differences between the method in this paper and existing technologies.**

**Our response:** We are grateful to the reviewer for his elaborate work to find out our weakness.We fully acknowledge the reviewer's comments and have revised Section 7.2 to elaborate on the theoretical innovations and clearly distinguish the fundamental differences between our methodology and existing techniques.

**Revised text:**

The core theoretical contribution of this paper lies in proposing and validating a “Full-Process Digital Twin with Tightly Coupled Physical Systems” architecture. Through the organic integration and closed-loop feedback between its automation execution and digital twin intelligence layers, this architecture overcomes the limitations of existing technologies.Its fundamental difference from current production technologies achieves a paradigm shift from “single-point automation optimization” to “end-to-end data-driven intelligence,” rather than merely replacing equipment or simply layering technologies.

The traditional production process is as follows: The injection molding machine first forms the base component of the smoke alarm. This is then manually sorted before being transported to the assembly stage. On the assembly line, workers assemble several metal parts with the base, followed by another round of manual sorting. Subsequent steps include manually folding cardboard boxes, packing assembled products into boxes, folding outer cartons, manually applying plastic edge sealing, bundling, and palletizing. The entire process requires 30 operators to meet production capacity demands.

The existing production line has undergone automation upgrades, significantly optimizing processes: After injection molding, base components are automatically unloaded by robotic arms and conveyed via conveyor belts to assembly equipment. Robots then pick up products for visual inspection. Following assembly of metal parts, a secondary visual inspection is conducted. Qualified products are then conveyed to automated packing equipment. A case opener automatically unfolds and positions the cardboard box, into which a robot automatically places the product. Subsequently, products sequentially travel via conveyor belt to a case sealer for primary and secondary sealing, then enter an edge sealer for automatic edge sealing. They are then automatically bundled by a strapping machine and finally palletized. The entire production line requires only one operator to manage auxiliary material replenishment for continuous operation.

It is precisely on this foundation of automation, combined with the deeper integration of digital twin technology, that we have achieved a leap from “equipment replacing human labor” to “data-driven intelligence.” This has resulted in significant differences from traditional production models and existing partial automation technologies across multiple dimensions.

(1) Core theoretical innovations

Herein, we propose the "Full-Process Digital Twin with Tightly Coupled Physical Systems" architecture. This work constructs a five-tier, closed-loop intelligent production framework encompassing: the Physical Production Layer, the Data Acquisition and Transmission Layer, the Virtual Simulation and Modeling Layer,the Intelligent Decision and Optimization Layer, and the Execution and Feedback Layer. This architecture faithfully mirrors all production elements (equipment, process, material, quality, personnel) and facilitates their real-time dynamic interaction. It directly addresses the critical limitations of traditional systems, the decoupling of physical entities from their virtual models and obstructed data flow,thus providing a solid theoretical basis for holistic process optimization and intelligent production control.

We established a data-driven predictive control model founded on full-link, multi-source heterogeneous time-series data, including injection molding temperature, pressure, assembly force, equipment vibration, and visual inspection features. A suite of multi-parameter coupled models for quality prediction and equipment health assessment is proposed and validated. This model proactively prevents quality issues, shifting control from a passive "detect-and-react" mode to an active "predict-and-prevent" mode. It also replaces "reactive" or "preventive" maintenance with data-driven predictive maintenance, thereby significantly boosting system stability and reliability.

We established a data-driven predictive control model founded on full-stream, multi-source heterogeneous time-series data, including injection molding temperature, pressure, assembly force, equipment vibration, and visual inspection features. A suite of multi-parameter coupled models for quality prediction and equipment health assessment is proposed and validated. This model proactively prevents quality issues, shifting control from a passive "detect-and-react" mode to an active "predict-and-prevent" mode. It also replaces "reactive" or "preventive" maintenance with data-driven predictive maintenance, thereby significantly boosting system stability and reliability.

We designed an adaptive scheduling mechanism tailored for flexible production. Through the virtual simulation layer of the digital twin system, an algorithm based on genetic algorithms and reinforcement learning is introduced to iteratively optimize process parameters and production schedules. This capability allows for rapid simulation of production flows across various product specifications, optimization of parameter sets, and automatic generation of the most efficient production plan. Consequently, it drastically cuts down on debugging time and costs in real-world changeover scenarios, directly tackling the inherent pain points of traditional automated lines: "inflexibility, high changeover costs, and poor market responsiveness."

(2) Fundamental differences from existing technologies

Compared to existing related technologies, the method proposed in this paper achieves fundamental breakthroughs in the following aspects:

Distinction from mere automation transformation. Existing mere automation transformations (such as simply introducing robotic arms or automated conveyor lines), while capable of replacing manual operations to a certain extent and improving production efficiency, essentially represent a "rigid" production mode. The production process lacks effective real-time monitoring and dynamic optimization capabilities, and the correlations between equipment status, process parameters, and product quality are difficult to deeply explore and utilize. The overall production system remains in a "black box" state, with decisions primarily based on experience. In contrast, our method, centered on the "Full-Process Digital Twin with Tightly Coupled Physical Systems," achieves complete "transparency" and "predictability" of the production process through digital twin technology. This elevates the system to the level of "data-driven intelligent decision-making," realizing a qualitative leap from "replacing human labor" to "globally optimizing the entire production chain."

Distinction from isolated digital twin applications.Currently, the application of digital twin technology in manufacturing is mostly confined to individual processes or single pieces of equipment,for instance, being used only for simulation and optimization of the injection molding process, or for health monitoring of a specific critical machine. Such isolated application modes result in “information silos” of data, where information from different stages cannot be effectively integrated or shared, making it difficult to form a holistic understanding or achieve system-wide optimization of the entire production process. In contrast, the method proposed in this paper extends digital twin technology across the entire workflow,from product molding and component assembly to quality inspection, packaging, and palletizing,encompassing the full sequence of “molding – assembly – inspection – packaging – palletizing.” This enables seamless data flow and deep integration across all stages. The virtual model can receive real-time feedback from the physical layer and dynamically adjust and optimize accordingly, thereby guiding the operation of the physical system in reverse and forming a complete closed-loop optimization cycle.

Distinction from traditional data-driven production.Traditional data-driven production systems primarily focus on collecting, storing, and performing basic statistical analysis of production data. Their main purpose is often limited to post-event traceability and report generation. A key limitation is their general lack of capability for virtual simulation, scenario modeling, and predictive analysis using the collected data.Consequently, the decision-making process in such systems remains heavily dependent on the experiential judgment of managerial personnel. This reliance on human intuition introduces subjectivity and limits proactive optimization.In clear contrast, our method fully utilizes the virtual simulation and modeling power of digital twin technology. We construct a virtual production system that maintains high fidelity with the physical world. This virtual environment allows for the simulation, testing, and optimization of various production scenarios without disrupting actual operations. The optimal strategies identified in this virtual space are then deployed directly to the physical system.This creates a continuous "trial-and-error – optimization" cycle grounded in virtual simulation. When integrated with machine learning algorithms, this cycle enables the self-optimization of process parameters, self-traceability of quality issues, and self-prediction of equipment failures. Ultimately, this approach achieves truly intelligent, autonomous operation and facilitates the continuous improvement of the production system.

(3) Quantitative validation and technical advantages (comparison between traditional production models and the method presented in this paper)

Based on the aforementioned theoretical innovations, the modern digital twin production line (technology-driven + data intelligence) has fundamentally transformed traditional production models.

Core limitations of traditional production models (labor-intensive):

Production efficiency, The entire manufacturing process relies heavily on manual labor, with over 15 major operations performed by hand. Production cadence is severely constrained by factors such as worker skill levels, labor intensity, and physical fatigue. This results in a lengthy overall production cycle per unit, creating significant bottlenecks for capacity expansion and limiting daily output to a low ceiling.

Labor costs, to meet basic production capacity requirements, the production line must be staffed with at least 30 operators. Calculated at a comprehensive monthly labor cost per person of approximately 6,000 yuan (including wages, social insurance, management, etc.), direct labor costs alone amount to 180,000 yuan per month. This represents not only a substantial ongoing expense but also comes with numerous challenges in recruitment, training, and management. As labor costs rise year after year, this burden will become increasingly heavy.

Quality costs, quality inspection relies entirely on manual visual screening, resulting in relatively high rates of missed defects and misidentifications. This keeps the product defect rate at a persistently high level (2%–5%). Not only does this directly lead to elevated rework costs and potential material waste, but it may also trigger customer complaints and damage the company's reputation. More critically, when quality issues arise, the lack of effective data recording and traceability makes it difficult to quickly pinpoint the root cause.

Management models, the production process resembles a “black box,” making it difficult for managers to gain real-time, accurate insights into actual conditions on the production floor. Management decisions primarily rely on experience, lacking scientific and precise data support, which results in inefficient production scheduling and resource allocation.

The practical implementation results of the methods described in this paper:

Production efficiency, automated equipment ensures 24/7 continuous operation at a fixed, high-speed cycle. The digital twin system further amplifies this gain by using real-time data and virtual simulation to dynamically optimize the entire process, achieving an efficiency boost of 400% to 600% compared to the conventional model. Process debugging and planning in the virtual environment have also reduced changeover time by over 50%, which in turn greatly increases production flexibility and enables a faster response to diverse market needs.

Labor costs, the entire complex production process requires only one employee to handle auxiliary material addition and monitor system operation status. Direct labor costs plummeted to approximately 6,000 yuan per month, achieving monthly savings of up to 174,000 yuan compared to traditional methods—a 96.7% reduction in labor expenses. The employee's role has also evolved from a traditional manual laborer to a modern technical administrator and system maintainer.

Quality control, the introduction of robotic vision systems enables two rounds of 100% comprehensive precision inspections, ensuring consistent product quality. Concurrently, digital twin technology monitors and records critical process parameters throughout production(such as injection molding temperatures and press-fitting forces)in real time, achieving full traceability of product quality. Should any quality issues arise, they can be swiftly traced to specific production stages, equipment parameters, or even raw material batches, enabling predictive quality control. Consequently, the product defect rate has been significantly reduced to below 0.1%.

Value-added benefits of the digital twin.Beyond the direct benefits outlined above, digital twin technology delivers substantial additional value.By virtually mapping physical production lines in real time, it enables real-time equipment monitoring and predictive failure detection. Predictive maintenance strategies based on data analysis have effectively reduced unplanned downtime by over 70%. By simulating and testing different combinations of process parameters, continuous optimization of production processes has increased overall equipment effectiveness (OEE) by 5% to 15%. The system supports remote monitoring and decision-making, significantly enhancing management efficiency and quality control capabilities, particularly for group-wide and cross-regional production management.

Comprehensive cost and return on investment.Although the initial investment for establishing a digital twin production line is relatively high-including sensors, IoT devices, data acquisition and processing systems, digital twin platform software, and related integration services-this investment is typically recovered within 2 to 4 years. This is achieved through drastic reductions in labor costs, quality-related costs, and equipment downtime losses. Beyond the payback period, the system continues to generate significant profit gains. Moreover, due to its high flexibility and scalability, the company is endowed with stronger capabilities for scalable expansion and enhanced market competitiveness.

The theoretical innovations and practical implementations in this study demonstrate a core value that goes beyond mere "human-to-machine substitution" through automation. More significantly, by constructing a "Full-Process Digital Twin with Tightly Coupled Physical Systems" architecture, we have achieved an integrated operational model in smoke alarm production for the first time. This model seamlessly combines "Digital Twin Mirroring," "Data Closed-Loop Driving," and "Predictive Intelligent Control" across the entire workflow—from product design and process planning to production execution and after-sales service.

This breakthrough addresses the common limitations of existing technologies, such as isolated point optimization, the virtual-physical disconnect, and rigid production structures. Consequently, it provides a replicable theoretical framework and a validated technical pathway for discrete manufacturing. It specifically facilitates the transition from traditional labor-intensive models to a modern "technology- and data-dual-driven" intelligent production paradigm, supported by quantitative evidence of its success.
